# Activation-induced cytidine deaminase an antibody diversification enzyme interacts with chromatin modifier UBN1 in B-cells

**DOI:** 10.1038/s41598-023-46448-7

**Published:** 2023-11-10

**Authors:** Ankit Jaiswal, Rajarshi Roy, Anubhav Tamrakar, Amit Kumar Singh, Parimal Kar, Prashant Kodgire

**Affiliations:** Department of Biosciences and Biomedical Engineering, Indian Institute of Technology, Indore, Madhya Pradesh 453 552 India

**Keywords:** Computational biology and bioinformatics, Immunology, Molecular biology

## Abstract

Activation-induced cytidine deaminase (AID) is the key mediator of antibody diversification in activated B-cells by the process of somatic hypermutation (SHM) and class switch recombination (CSR). Targeting AID to the Ig genes requires transcription (initiation and elongation), enhancers, and its interaction with numerous factors. Furthermore, the HIRA chaperon complex, a regulator of chromatin architecture, is indispensable for SHM. The HIRA chaperon complex consists of UBN1, ASF1a, HIRA, and CABIN1 that deposit H3.3 onto the DNA, the SHM hallmark. We explored whether UBN1 interacts with AID using computational and in-vitro experiments. Interestingly, our in-silico studies, such as molecular docking and molecular dynamics simulation results, predict that AID interacts with UBN1. Subsequently, co-immunoprecipitation and pull-down experiments established interactions between UBN1 and AID inside B-cells. Additionally, a double immunofluorescence assay confirmed that AID and UBN1 were co-localized in the human and chicken B-cell lines. Moreover, proximity ligation assay studies validated that AID interacts with UBN1. Ours is the first report on the interaction of genome mutator enzyme AID with UBN1. Nevertheless, the fate of interaction between UBN1 and AID is yet to be explored in the context of SHM or CSR.

## Introduction

Antibody diversity is one of the key features of B-cells that assists in nullifying pathogens in humans. B-cells diversify their antibody repository from a handful of immunoglobulins (Ig) genes via VDJ recombination, somatic hypermutation (SHM), and class switch recombination (CSR). VDJ recombination occurs in an antigen-independent manner, whereas SHM and CSR occur in an antigen-dependent manner. Activation-induced cytidine deaminase (AID) is the initiator of SHM and CSR in the activated B-cells. An AID-introduced point mutation in the variable region of Ig genes leads to affinity maturation, whereas in switch regions results in immunoglobulin (Ig) isotype switching^1^. AID being a deleterious enzyme, its activity is predominantly confined to the Ig locus. However, AID can be off-targeted to non-Ig genes in transcriptionally active B-cells, especially to proto-oncogenes, resulting in dysregulation of proto-oncogenes and chromosomal translocation. Strikingly, aberrant expressions of AID are reported in infection as well as various types of B-cell lymphoma^2^.

**Figure 1:**
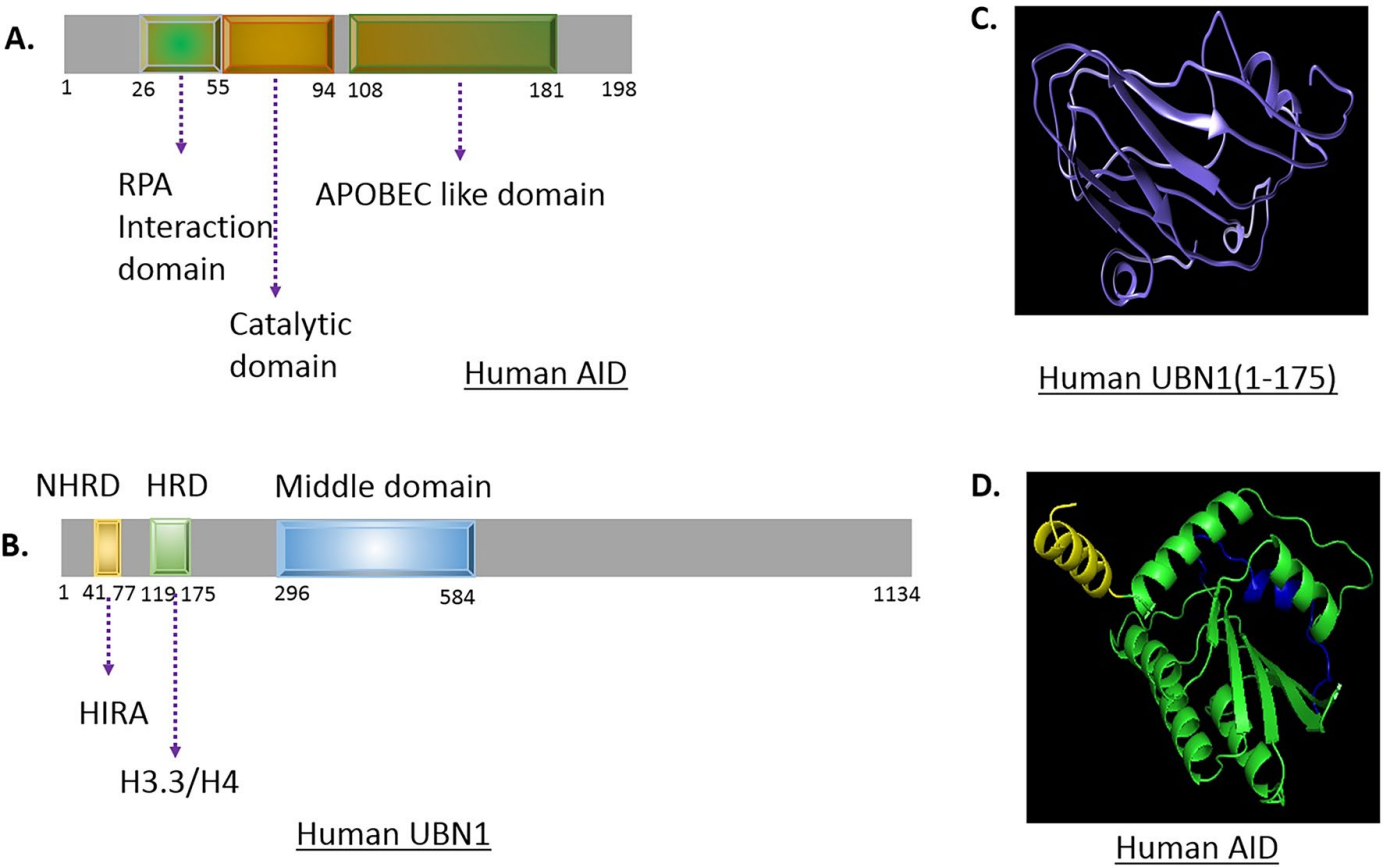
(**A**) The structural domain of human AID. (**B**) The structural domain of UBN1. The NHRD domain interacts with HIRA, and the HRD domain interacts with H3.3/H4. (**C**) UBN1 structure predicted by I-TASSER. (**D**) AID structure as predicted by alphafold2 (AF-Q9GZX7-F1).

During the early years of discovery, AID was thought to be an RNA editing enzyme. AID protein was discovered in 1999, encoded by the AICDA gene locus in humans^3^. Further, studies unravel AID's structure, function, and the mechanism of action. The AICDA gene encodes AID protein in humans and consists of five exons. AID is mainly composed of four domains, namely the N-terminal domain, catalytic domain, APOBEC domain, and C-terminal domain (Fig. 1A). N-terminal and C-terminal domains of AID are required for SHM and CSR, respectively^4^. AID is a shuttling protein that moves in and out of the nucleus due to the presence of a strong nuclear export signal and a weak nuclear localization signal, resulting in its predominant localization in the cytosol^5^. Moreover, AID’s predominant localization in the cytoplasm limits its activity and accessibility to transcriptionally active DNA^5^. Biochemical studies on purified AID protein revealed that it binds to single-stranded DNA and preferentially deaminates cytosine residue in AID hotspots WR**C** (W = A/T, R = A/G) motif in contrast to another region at Ig locus^6, 7^. Interestingly, despite having significant differences in the sequence of AID among species, AID showed a higher affinity for binding to DNA^8^. AID's low catalytic rate and strict regulation at transcriptional, post-transcriptional and post-translational levels prevent its harmful activity.

Understanding the targeting of AID to Ig genes and identifying its interaction partners are the areas of extensive research in the last two decades. Even before the discovery of AID, Betz et al.^9^ illustrated the significance of an intronic enhancer that assists in maintaining a high mRNA level essential for SHM. Subsequently, Peter et al.^10^ demonstrated that the SHM of Ig genes directly depends on the transcription initiation. Transcription elongation, RNA PolII pausing, and termination^11^ are also required for AID action. As transcription through DNA is directly linked with nucleosome architecture and composition, AID accessibility to single-stranded DNA directly depends on the action of the chromatin remodelling complex and histone chaperone's complex. Interestingly, the Facilitates Chromatin Transcription Complex (FACT)^12^ and HIRA chaperone complex are enriched at the Ig locus and are required for AID-mediated SHM^13^. FACT and HIRA are responsible for the deposition of histone variant H3.3 at the Ig locus in a replication-independent manner leading to the opening of chromatin at the Ig locus. Besides, transcription, FACT, and HIRA complex, AID targeting to Ig locus also requires its interaction with transcriptional machinery components such as RNA PolII, Spt5^14^, and replication protein A^15^ (RPA). Additionally, AID interactions with splice regulators and factors like SRSF1-3^16^, CTNNBL1^17^, and PTBP2^18^ are well documented.

Ubinuclein 1 (UBN1) is a part of the HIRA chaperon complex that also consists of HIRA, Calcineurin-binding protein 1 (CABIN1), and Anti-silencing factor 1a (ASF1a). HIRA chaperon complex in association with H3.3 is required for gene activation^19^ as well as it is responsible for gene silencing^20^. UBN1 plays a key role in the deposition of H3.3 at gene regulatory elements in ES cells of the mouse^21^. UBN1 domains comprised of N-terminal Hpc2-related domain (NHRD) domain, HRD domain (Fig. 1B), and middle domain. UBN1 interacts with HIRA through the NHRD domain and H3.3 through the HRD domain (Fig. 1B). Interestingly, HIRA chaperon complex specificity to H3.3 is imparted through UBN1 and is responsible for de novo deposition of H3.3 at the Ig locus which is also the hallmark of SHM^12^. Remarkably, SRSF1-3, a splicing regulator which is necessary for SHM^22^, also upregulates the expression of UBN1 in DT40 SRSF1-3 reconstituted cell lines^23^, which highlights the significance of UBN1 in SHM.

Zheng et al. reported that RPA interaction with HIRA protein, a member of the HIRA chaperon complex, and H3.3 is crucial for HIRA-mediated, H3.3 deposition at gene regulatory elements^24^. Incidentally, an earlier study showed that RPA also interacted with AID in activated B-cells^15^. In brief, AID interaction with RPA^15^ is well established, and RPA is also reported to interact with the HIRA chaperon complex’s HIRA protein^24^. In this study, we explore whether there is any interaction of AID with UBN1, a member of the HIRA chaperone complex. In our study, we employed in-silico studies like molecular docking and molecular dynamics (MD) simulation, co-immunoprecipitation, in-vitro pull down assay as well as double immunofluorescence assay along with proximity ligation assay to demonstrate the interaction between AID and UBN1. This study unfolds yet another AID interacting partner, UBN1. However, the AID-UBN1 interaction and its significance in the context of SHM or CSR still need to be explored.

## Materials and methods

### Structural modelling of UBN1 and docking analysis

Homology modelling of UBN1 was performed using the I-TASSER web server^25, 26^ to obtain the 3D structure from the amino acid sequence retrieved from Uniport ID (Q9NPG3). Models were generated using the threading approach, and the best model was selected based on the C score. For AID, we downloaded the already predicted structure from the Alpha fold database (AF-Q9GZX7-F1)^27^. The UBN1-AID complex was formed by the docking protocol using the Rosetta online server^28^. Further, the docked complex was selected according to the docking score and further subjected to molecular dynamics (MD) simulation.

### Molecular dynamics simulation

The UBN1-AID complex was subjected to 150 ns MD simulation using the *pmemd.cuda* module of AMBER18 suite^29^. Amber ff14SB forcefield was used to simulate the protein–protein complex^30^. The complex was neutralized with an appropriate number of ions and solvated into an octahedron water box with TIP3P water molecules^31^. SHAKE algorithm was used to restrain the bonds involving hydrogen atoms to keep the timestep of simulation 2 fs^32^. The long-range interaction was estimated using the particle mesh Ewald (PME) method with a non-bonded cut-off of 10 Å^33^. The temperature was kept constant at 300 K using a Langevin thermostat with a collision frequency of 2 ps^−1^^34^. Similarly, the pressure of the system was also controlled using Berendsen's Barostat. Complexes were subjected to two-step minimizations followed by a stepwise heating phase and equilibrium simulation before the final production run. We performed a 100 ns long equilibrium simulation to remove any structural clashes and stabilized the protein–protein complex. A detailed description of the simulation protocol was already discussed in our previous works^35, 36^. Finally, we performed a 50 ns production run under the NPT ensemble. Trajectory analysis was conducted using the *cpptraj* module of AmberTools^37^.

### Binding free energy analysis

The binding free energy was calculated using the molecular mechanics/Poisson Boltzmann surface area (MM/PBSA) scheme. The following equations were employed to estimate the binding free energy^38^

1 $${\Delta G}_{binding}= {G}_{complex}-({G}_{protein1}+ {G}_{protein2})$$

where the energy term further subdivided into

2 $$G={E}_{vdW}+{E}_{ele}+{G}_{pol}+{G}_{np}$$

${E}_{vdW}$ and ${E}_{ele}$ represent the van der Waals and electrostatic interaction energies. The solvation term, ${G}_{pol}$, indicates the electrostatic solvation free energy estimated using the Poisson Boltzmann equation and ${G}_{np}$ represents the non-polar solvation. Configurational entropy was avoided in our current study as we are interested only in the relative binding free energy and due to the higher computational cost. We have used 2500 configurations obtained from the last 50 ns trajectory for the MM/PBSA calculation. Residue-wise contribution of binding free energy was also estimated using the molecular mechanics Born generalized surface area (MM/GBSA) scheme^39^.

### Cell culture

Raji cells (procured from NCCS, Pune) were grown in RPMI 1640 medium consisting of 10% FBS, 50 μM 2-mercaptoethanol, 2 mM glutamine, 100 μg/ml penicillin G, 50 μg/ml streptomycin at 37 °C, 5% CO_2_. DT40 ψVKO cells were a gift of Dr. H. Arakawa and Dr. J.M. Buerstedde (Institute of Molecular Radiology, Neuherberg, Germany) to Prof. U Storb, which were grown in similar media as Raji cells, supplemented with 1% chicken serum at 39.5 °C, 5% CO_2_.

### Co-localization of AID and UBN1

For performing a co-localization assay, 5 × 10^6^ DT40 ψV KO and Raji cells were washed 3 times in 1X PBS. Subsequently, cells were fixed in 4% paraformaldehyde in 1X PBS for 20 min. Cells were permeabilized in 0.1% triton X in 1X PBS for 20 min and blocked in 5% BSA in PBS-T for 30 min. Furthermore, cells were incubated with rabbit anti-human UBN1 (Sigma Anti-UBN1 antibody produced in rabbit, Cat. # HPA069045) and mouse anti-human AID (Thermo Fischer Scientific anti-AID produced in mouse, Cat. # 39-2500) (1:100 dilutions) overnight at 4 °C in 1X PBS supplemented with 2.5% BSA. Again, cells were washed 3 times with 1X PBS-T followed by incubation with anti-rabbit Alexa flour 594 (Thermo Fischer Scientific anti-rabbit produced in donkey, cat # A21207) and anti-mouse Alexa flour 488 (Thermo Fischer Scientific anti-mouse produced in goat, cat # A11001) (1:500 dilutions) secondary antibodies in 2.5% BSA for 60 min. Cells were washed 3 times and stained with DAPI nuclear stain for 15 min. Lastly, cells were resuspended in 1X PBS and mounted on the slide, kept for drying at 37 °C, and high-resolution images were captured via confocal microscopy (Olympus FV3000).

### Co-immunoprecipitation of AID and UBN1

For CoIP from whole-cell extract, 2 × 10^7^ cells were washed 3 times with 1X PBS, incubated with 2 mM DSP (Thermo Scientific) in PBS for 2 h at 4 °C for cross-linking, and terminated with 20 mM Tris–HCl pH 8.0. Subsequently, cells were lysed in lysis buffer (50 mM Tris–Cl, pH-8.0, 150 mM NaCl, 1.0% NP-40, Protease inhibitor cocktail supplemented with 1 μg/ml DNaseI). Furthermore, lysate DNA was sheared by passing through a 22-gauge needle multiple times. Lysate supernatants were incubated with either anti-AID mAB (Invitrogen) or anti-UBN1 polyclonal antibody (Sigma-Aldrich) (1:100 dilutions) overnight at 4 °C on a rota-shaker. Later, Protein-A agarose beads were washed and equilibrated in equilibration buffer (20 mM Tris–Cl pH-8.0, 5 mM MgCl_2_, 5 mM MnCl_2_, 150 mM NaCl, and 1.0% NP-40). Subsequently, lysates were incubated with protein-A agarose beads, kept on a rota-spin for 2 h at 4 °C, and pelleted by centrifugation. Pellets were washed three times in washing buffer (20 mM Tris–Cl pH-8.0, 5 mM MgCl_2_, 5 mM MnCl_2_, 750 mM NaCl, and 1.0% NP-40) with 5 min incubations between spins. Lastly, pull-down proteins were eluted in elution buffer (120 mM Tris–Cl, pH 6.8, 1.0% SDS, at room temperature for 30 min on a shaker) and boiled at 95 °C for 5 min. Subsequently, a western blot was performed, and membranes were first incubated with anti-UBN1 or anti-AID. Finally, the blot was incubated with either Goat anti-mouse IgG-HRP, HS201 (Trans Bionovo), or goat anti-rabbit HRP (GeNei) secondary antibody, and an image was taken.

### Pull down assay

His-MBP-UBN1^1-175^, His-MBP-AID, and His-MBP were expressed in *E. coli* and purified using standard Ni-NTA beads using manufacture guidelines (Bio-Rad Nuvia IMAC resin). Subsequently, all three proteins were buffer exchanged against 200 mM NaCl, 25 mM Tris–Cl pH 8.0, 10% glycerol, and 0.5 mM TCEP. Further, equimolar of His-MBP-UBN1^1-175^, His-MBP-AID, and His-MBP were separately incubated with 1.5 mg whole cell lysate of Raji or DT40ψV KO (lysis buffer contains 1X PBS, 1% NP-40, protease inhibitor cocktail and 1 mM beta-mercaptoethanol) in 1XPBS with mild agitation for 6 h/4 °C for binding. Further, 80 μl of pretreated Ni-NTA beads were added to each tube and incubated for 2 h/4 °C. Subsequently, beads were washed thrice with 1 ml wash buffer (200 mM NaCl, 10 mM Tris–Cl pH 8.0, 50 mM imidazole, 1 mM beta-mercaptoethanol) by centrifugation at 800 g. Protein-containing Ni-NTA beads were eluted in a wash buffer supplemented with 500 mM imidazole. Finally, samples were subjected to SDS-PAGE followed by western blotting and analysed either by anti-AID mAb or anti-UBN1 pAb.

For in-vitro His-MBP-hAID pull-down experiments, a new clone MBP-Flag-UBN1^1-175^ (without His-tag) was created. Equimolar concentrations of His-MBP-hAID and His-MBP were mixed with MBP-Flag-UBN1^1-175^, separately, in the binding buffer (200 mM NaCl, 10 mM Tris–Cl pH 8.0, 10% glycerol, 0.1% tween 20, 1 mM βME supplemented with 20 mM imidazole), kept for 6 h/4 °C  with mild agitation. Subsequently, 40 μl Ni-NTA beads were added in each tube and kept for 2 h/4 °C, washed thrice with binding buffer supplemented with 50 mM imidazole and eluted with binding buffer supplemented with 500 mM imidazole, detected with anti-flag antibody (Cat. # F1804 monoclonal anti-flag M2 antibody produced in mouse, Sigma-Aldrich). Further, for pull-down studies with His-MBP-Flag-UBN1^1-175^, a new clone of MBP-hAID (without His-tag) was created. Similarly, in His-MBP-Flag-UBN1^1-175^ pull-down experiments, equimolar quantities of His-MBP-Flag-UBN1^1-175^ and His-MBP were mixed with MBP-hAID and proceeded as mentioned for His-MBP-hAID pull-down protocol mentioned above. Finally, western blotting was performed using anti-AID antibody.

### Proximity ligation assay

PLA was carried out as suggested by Duolink in situ Red PLA kit Mouse/Rabbit (Sigma-Aldrich, DUO92101) manual. For PLA, 3 × 10^6^ Raji cells were taken and processed in a similar fashion as mentioned in the co-localization assay till the overnight primary antibodies incubation. Further, cells were washed twice with wash buffer-A and incubated with oligonucleotides conjugated secondary antibodies (PLA Probes anti-mouse MINUS and PLA Probes anti-rabbit PLUS) for 60 min at 37 °C in a humidity chamber. Subsequently, cells were washed twice with wash buffer and incubated with a ligation mixture for 30 min at 37 °C in a humidity chamber. Cells were washed twice with wash buffer A and centrifuged at 4 °C for 5 min. Further, a polymerase amplification mixture was added and incubated for 120 min at 37 °C. Cells were again washed twice with wash buffer-B at 4 °C for 10 min, and the final wash was with 0.01X wash buffer-B for 1 min. Finally, DAPI with the mounting medium was directly added to the cells, mounted onto the slides, and left overnight for drying at 4 °C, scanned, and images were captured using a confocal laser scanning microscope (Olympus, FV1200MPE). Similarly, PLA assay was performed with DT40ψV KO cells as suggested by Duolink in situ-detection reagents far red (Sigma-Aldrich, DUO92013) manual.

## Results

### In-silico estimation of UBN1-AID interaction

**Figure 2:**
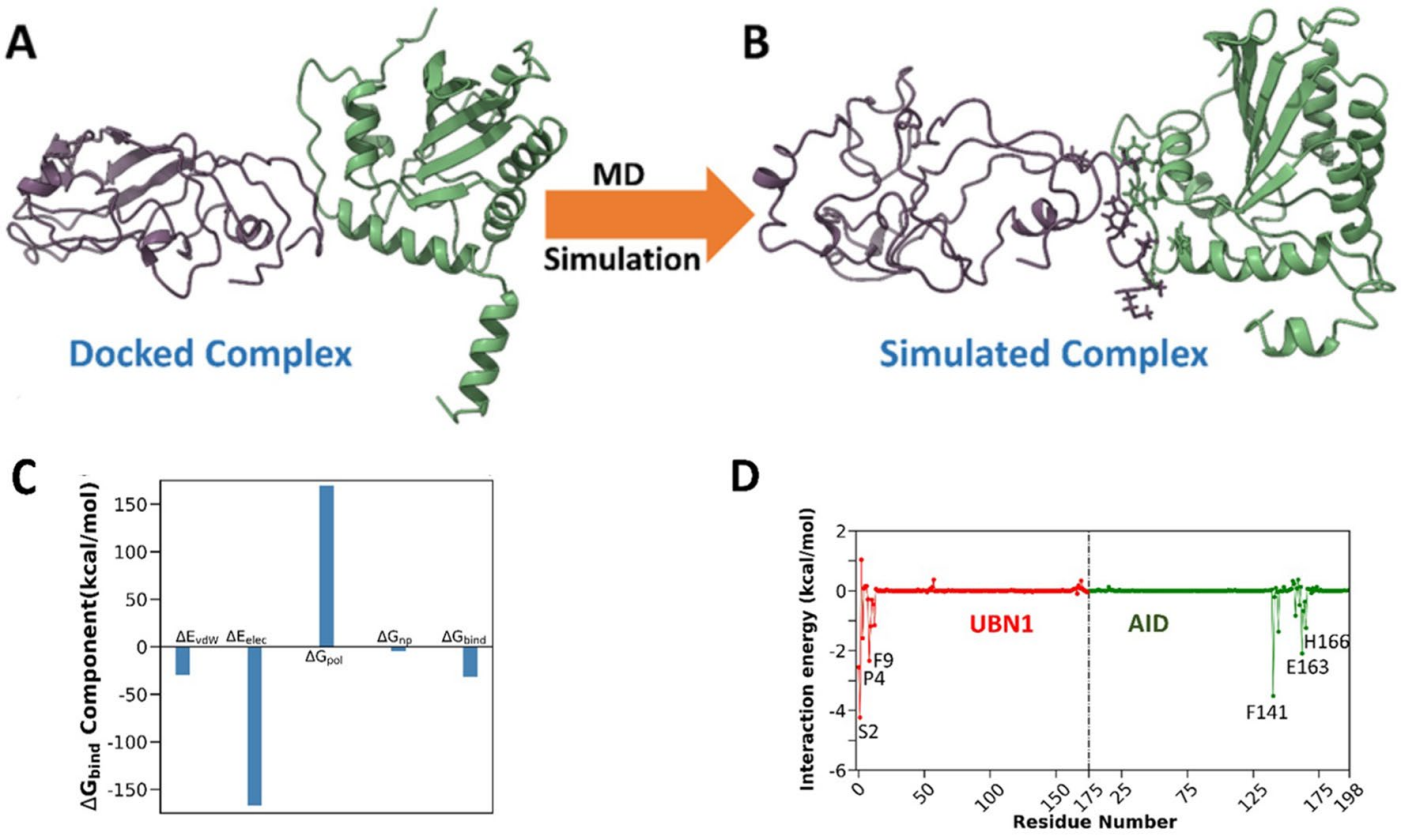
(**A**) UBN1 and AID complex after docking, (**B**) UBN1 and AID complex after MD simulation. UBN1: Violet, AID: Green, (**C**) Binding free energy and its component for UBN1 and AID complex, (**D**) Residue-wise contribution of binding free energy. Key residues are mentioned in the plot.

AID is a key mediator of the antibody diversity in the activated B-cells. AID actions and targeting is an orchestrated event that requires the cross-talk between numerous proteins and cis-acting elements. Interestingly, UBN1 is also a crucial member of the chromatin modifier complex that cross-talks with one of the AID interacting partners, RPA^24^. In this study, we wanted to explore whether AID and UBN1 interact with each other or not, which may have a role in antibody diversity. Furthermore, homology modeling and molecular docking study were used to shed light on the molecular interaction between UBN1-AID. Due to very less sequence similarity, we modelled only the N-terminal domain (1-175) of UBN1 using the i-TASSER web server (Fig. 1C). On the other hand, the modelled structure of AID was already available in the alpha-fold webserver (Fig. 1D). The Ramachandran plot was calculated for modelled UBN1 to validate the structure. A molecular docking study was conducted for the modelled structure using Rosetta v.3.2 using the online Rosie web server^40^. Among the top ten models, the lowest energy-minimized structure was used for the input of the MD study. Initial inspection of UBN1-AID showed that interaction was mainly driven through the initial N-terminal residues of UBN1. The three-dimensional conformation of the docked complex and its conformational change after the MD run are shown in Fig. 2A and B. Conformational change upon MD indicates the stability of the binding.

An MD simulation was conducted to estimate the molecular mechanism of the UBN1 and AID. A 100 ns long equilibrium stage was undertaken to stabilize the complex as well as UBN1 and AID. The reason behind the long equilibrium step is to stabilize the model complex prior to the binding free energy calculation. The final production run of 50 ns shows the stable conformation of for complex as well as in both the protein as demonstrated from the time evolution of root mean square deviation (RMSD) (Fig. S1A). Further, to estimate the residual fluctuation, we estimate the root mean squared fluctuation (RMSF) (Fig. S1B), which shows higher flexibility of UBN1 compared to AID. However, the interface residues of UBN1 (initial N-terminal residues) show lesser fluctuation indicating a stable binding. Also, to estimate the stability of the binding between UBN1 and AID, the centre of mass distance was calculated, which remains stable in the due course of our simulation (Fig. S1C). This was also supported by the stable 2–3 hydrogen bonds between the UBN1 and AID through the simulation length (Fig. S1D).

To estimate the binding in more detail, molecular mechanics Poisson Boltzmann surface area (MM/PBSA) was used throughout the MD trajectory and shown in the Supporting Information (Table S1). Different components of the binding free energy are also shown graphically in Fig. 2C and Table S1. It is evident from Table S1, that intermolecular van der Waals interaction (*E*_vdW_), electrostatic interaction (*E*_elec_), and non-polar solvation (*G*_np_) favoured the interaction, while polar solvation (*G*_pol_) disfavoured the binding. The binding free energy between the proteins is − 31.18 kcal/mol (Fig. 2C and Table S1). The interaction pattern shows a higher contribution from electrostatic components (− 167.05 kcal/mol), however, this completely cancels due to an increase in the polar solvation contribution (169.88 kcal/mol). So, the main interaction was governed due to the van der Waals contribution (− 29.90 kcal/mol) (Fig. 2C and Table S1). Furthermore, this is supported by our Ligplot^+^ analysis, which shows a greater number of hydrophobic contacts (Fig. S2). Also, to evaluate the key residues in the binding process, the decomposition of binding free energy was calculated (Fig. 2D). The key residues in the binding process are S2, P4, and F9 for UBN1 and F141, E163, and H166 for AID. This indicates the binding residues mainly reside around the N-terminal and C-terminal of UBN1 and AID, respectively. As the lack of sequence similarities, we restrict our model to the N-terminal part of UBN1. So, the findings of the computational investigation can shed light on the molecular mechanism of the interactions. This will be one of the first efforts to quantify the interaction pattern between UBN1 and AID**.**

### Interaction of AID with UBN1

**Figure 3:**
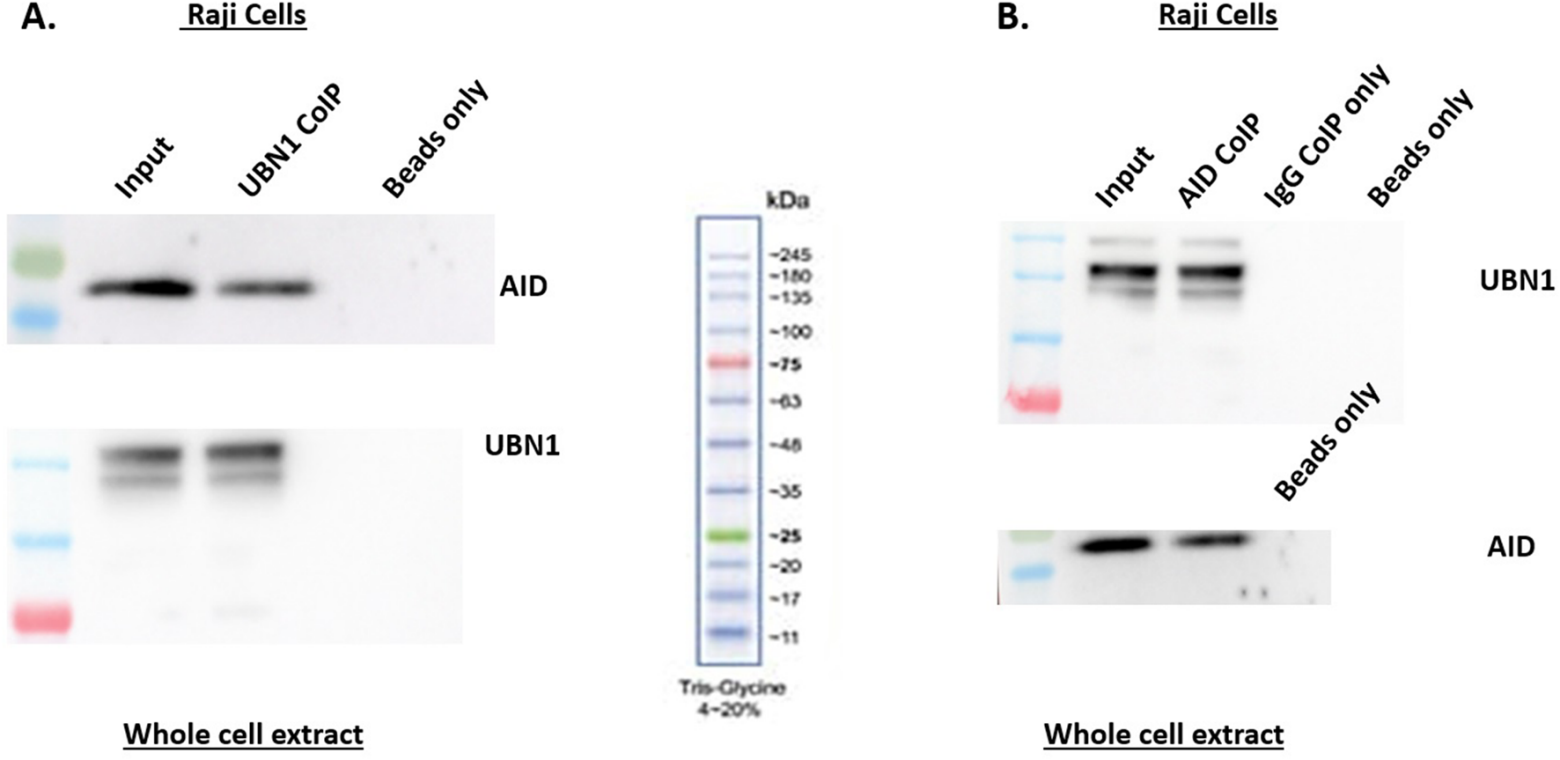
AID interaction with UBN1 in Raji cells. (**A**) Co-immunoprecipitation of UBN1 from whole cell lysate was performed from Raji cells using anti-UBN1 pAb and analyzed by western blotting using protein anti-AID mAb and anti-UBN1 pAb. (**B**) Co-immunoprecipitation of AID from whole cell lysate of Raji cells using anti-AID mAb and analyzed by western blotting using anti-UBN1 pAB and anti-AID mAb**.**

**Figure 4:**
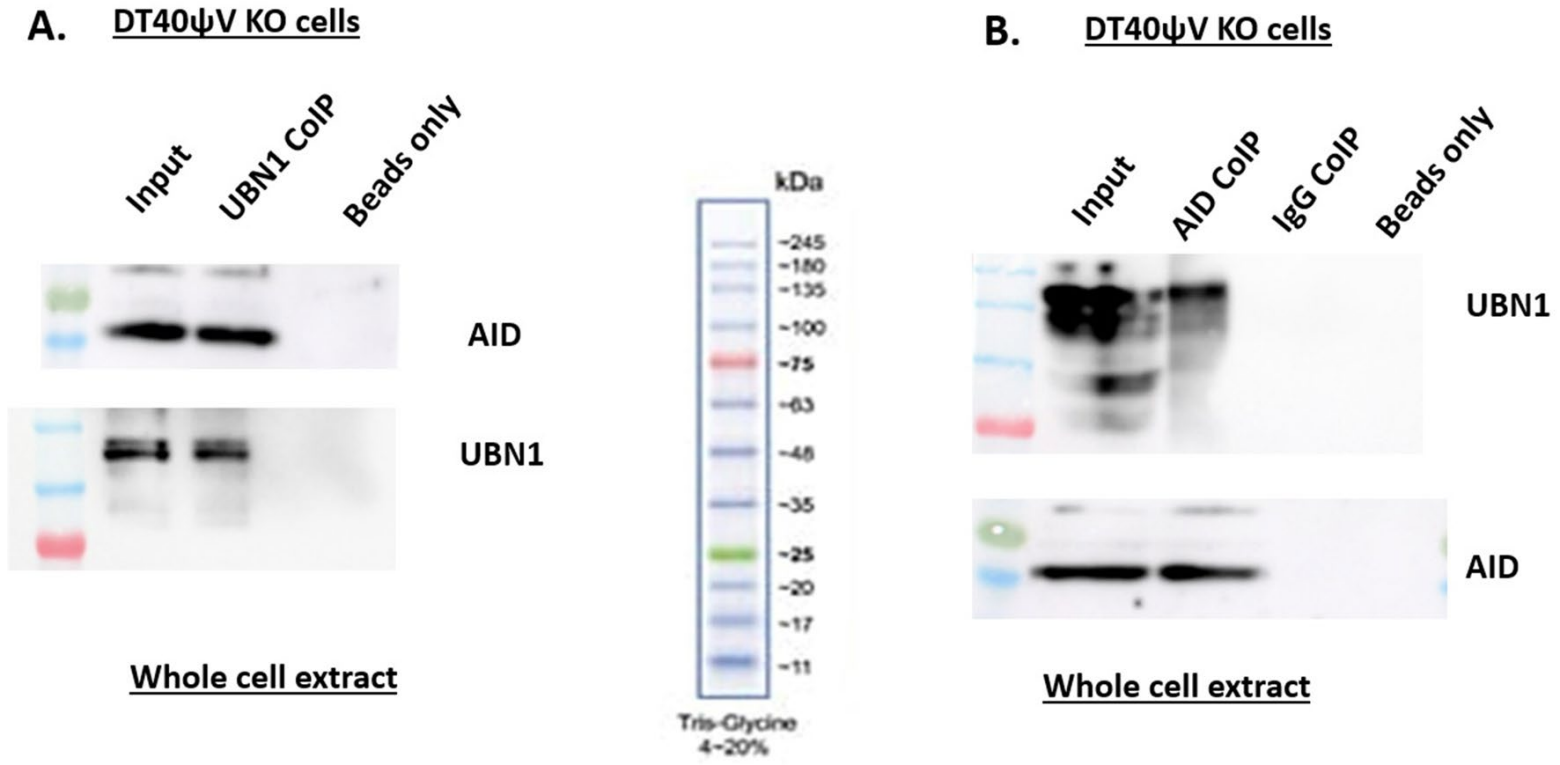
AID interaction with UBN1 in DT40ψV KO cells. (**A**) Co-immunoprecipitation of UBN1 from whole cell lysate was performed from DT40ψV KO using anti-UBN1 pAb and analyzed by western blotting using protein anti-AID mAb and anti-UBN1 pAb. (**B**) Co-immunoprecipitation of AID from whole cell lysate of DT40ψV KO cells using anti-AID mAb and analyzed by western blotting using anti-UBN1 pAB and anti-AID mAb.

In-silico studies predicted that UBN1 interacts with AID. We performed co-immunoprecipitation experiments to validate further whether UBN1 AID interaction happens in B-cells. In the co-immunoprecipitation assay, we pulled down AID using an anti-AID antibody from human Raji cells and chicken DT40 ψVKO cells and detected using an anti-UBN1 antibody (Figs. 3B and 4B, Figs. S3 and S4). As anticipated, an AID interacting partner UBN1 was detected in both cell lines. Similarly, we pulled down UBN1 using an anti-UBN1 antibody in Raji and DT40ψV KO and probed with an anti-AID antibody (Figs. 3A and 4A, Fig. S3 and S4). Likewise, UBN1 interacting partner AID was detected. So, based on our co-immunoprecipitation, we report that AID interacts with a member of the HIRA chaperon complex UBN1 in B-cells. Interestingly, as AID interacts with UBN1, how this interaction influences the SHM or CSR at the Ig locus needs to be explored further. Moreover, a co-immunoprecipitation assay was also performed from DT40 AID KO cell lysate and pulled down using anti-UBN1 and WB anti-AID and vice versa to eliminate any non-specific pull-down. As anticipated, no signal (Fig. S5) was detected in the Co-IP sample, suggesting that pull down in DT40ѰV KO cells was specific. Further, pull down experiment using an anti-HIRA antibody (anti-HIRA cat # SAB4503047, Millipore-Sigma) as well as anti-ASF1a (anti-ASF1a cat # ABE149, Sigma) to identify any association of AID with other members of HIRA chaperon complex. Interestingly, AID was not found to be associated with either HIRA or ASF1a (Figs. S6, S7 and S8), suggesting that AID is not interacting with HIRA or ASF1a.

**Figure 5:**
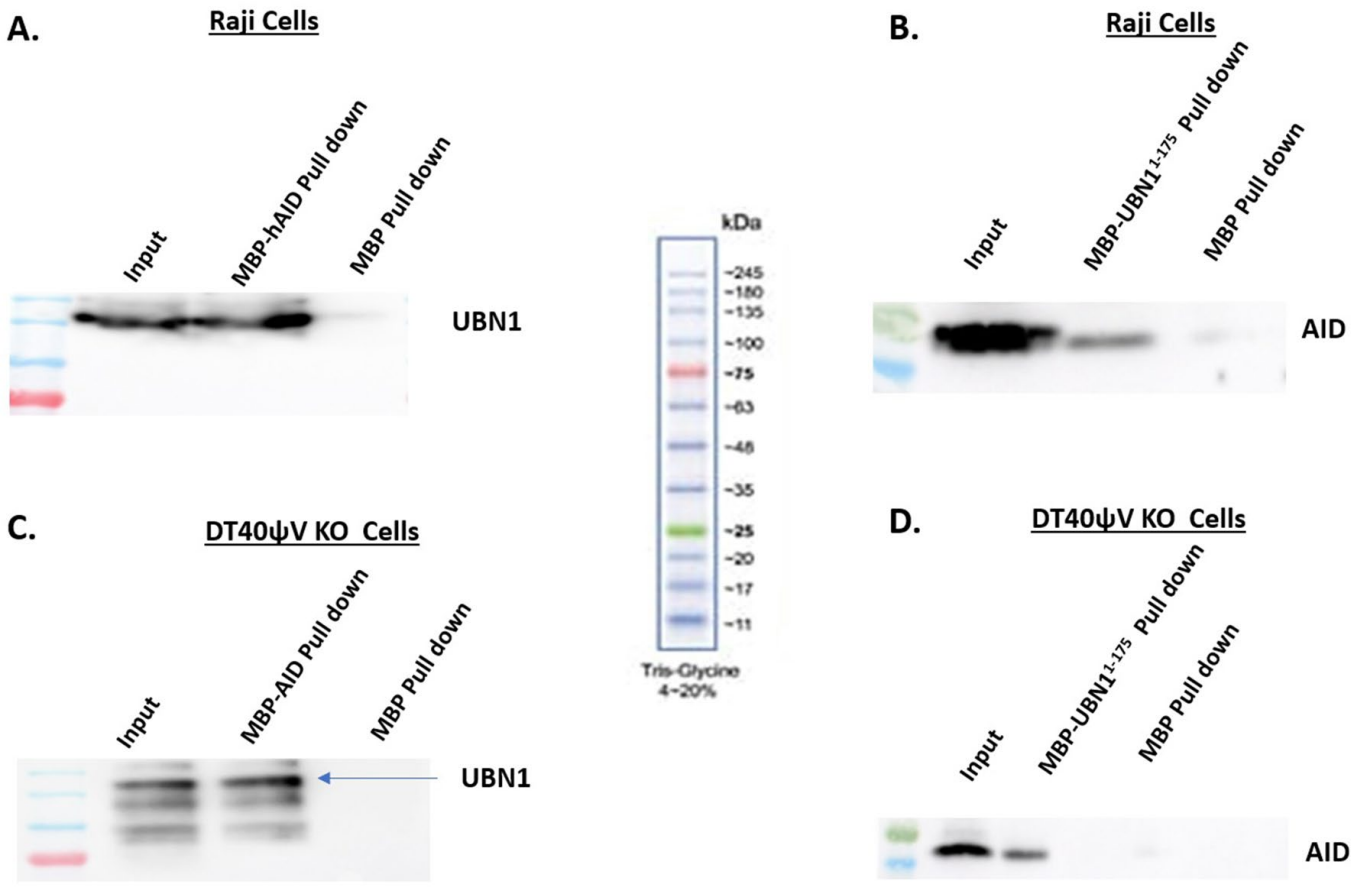
His-tagged pull-down assay for AID interaction with UBN1 in Raji and DT40ψV KO cells. (**A**) His tagged MBP-AID or MBP were immobilized on Ni-NTA beads followed by incubation with Raji cell lysate and analysed by western blotting using anti-UBN1 pAb. (**B**) His tagged MBP-UBN1 or MBP were immobilized on Ni-NTA beads followed by incubation with Raji cell lysate and analysed by western blotting using anti-AID mAb. (**C**) His tagged MBP-AID or MBP were immobilized on Ni-NTA beads followed by incubation with DT40ψV KO cell lysate and analysed by western blotting using anti-UBN1 pAB. (**D**) His tagged MBP-UBN1 or MBP were immobilized on Ni-NTA beads followed by incubation with DT40ψV KO cell lysate and analysed by western blotting using anti-AID mAB.

Further, to validate that AID interaction with UBN1 is specific, we performed an in vitro pull-down assay where either purified AID or UBN1^1-175^ was incubated with cell lysate. We incubated purified His-MBP-UBN1^1-175^ with Raji and DT40ψV KO cell lysate and pulled down using Ni-NTA beads, and AID was detected in the pull-down sample (Fig. 5B and D, Fig. S9B and D). Similarly, His-MBP-AID protein was incubated with Raji and DT40ψV KO cell lysate and pulled down using Ni-NTA beads, and UBN1 was detected (Fig. 5A and C, Fig. S9A and C). As anticipated, His-MBP-hAID was found to interact with UBN1 and vice-versa. Moreover, His-MBP was used as a control to eliminate any possibility of purification and solubilization tag interacting with UBN1 or AID. Additionally, no band was observed in the control sample, which suggests the specific interaction between AID and UBN1.

**Figure 6:**
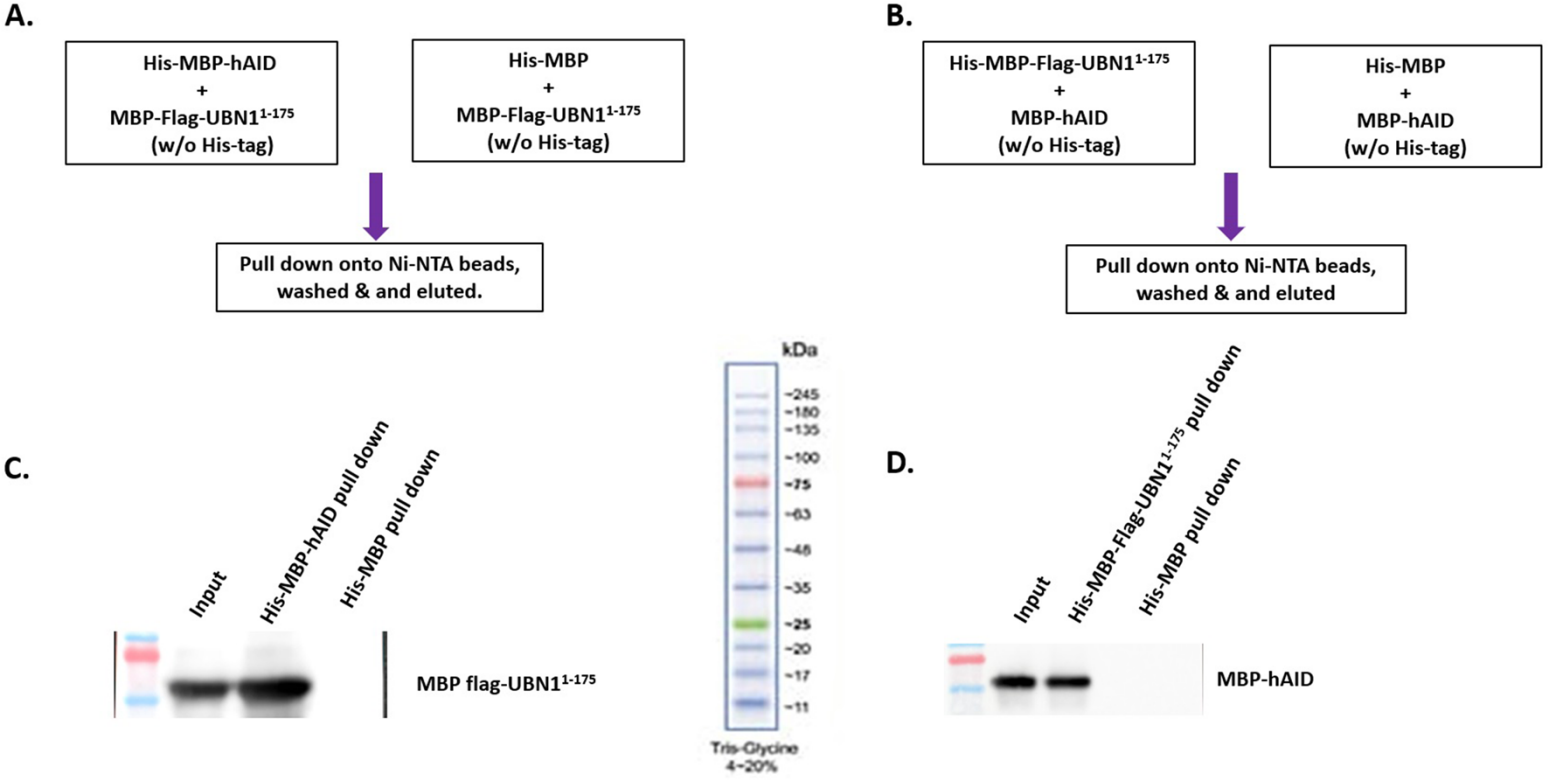
In-vitro pull-down assay using purified protein to detect the interaction between UBN1 and hAID. (**A**, **B**) represent the strategy used for pull-down in (**C**, **D**), respectively. (**C**) MBP-hAID (w/o His tag) was added to His-MBP-Flag-UBN1^1-175^ and His-MBP, followed by pull-down using Ni-NTA beads and detected by western blotting using anti-AID mAb. (**D**) MBP-Flag-UBN1^1-175^ (w/o His-tag) was added to His-MBP-hAID and His-MBP, followed by pull-down using Ni-NTA beads and detection by western blotting using anti-Flag-mAb.

Moreover, to confirm AID interaction with UBN1, we performed a pull-down assay using purified His-MBP-hAID, His-MBP, His-MBP-Flag-UBN1^1-175^, MBP-hAID (without His-tag) and MBP-Flag-UBN1^1-175^ (without His-tag). We incubated His-MBP-hAID with MBP-Flag-UBN1^1-175^ and pulled it down using Ni-NTA beads, and MBP-Flag-UBN1^1-175^ was detected using anti-flag Ab (Fig. 6A and C, Fig. S10A). Similarly, His-MBP-Flag-UBN1^1-175^ was incubated with MBP-hAID and pulled down using Ni-NTA beads, and MBP-hAID was detected using an anti-AID antibody. Furthermore, no signal was detected in His-MBP control neither in His-MBP-hAID nor His-MBP-Flag-UBN1^1-175^ pull down (Fig. 6B and D, Fig S10B). As anticipated, our in-vitro pull-down assay showed AID interacts with UBN1 and vice versa. Thus, similar to our co-immunoprecipitation assay, the pull-down assay using cell lysate, as well as the pull-down assay with purified proteins, strongly suggest that AID is interacting with UBN1.

### Co-localization and proximity ligation assay of AID with UBN1

**Figure 7:**
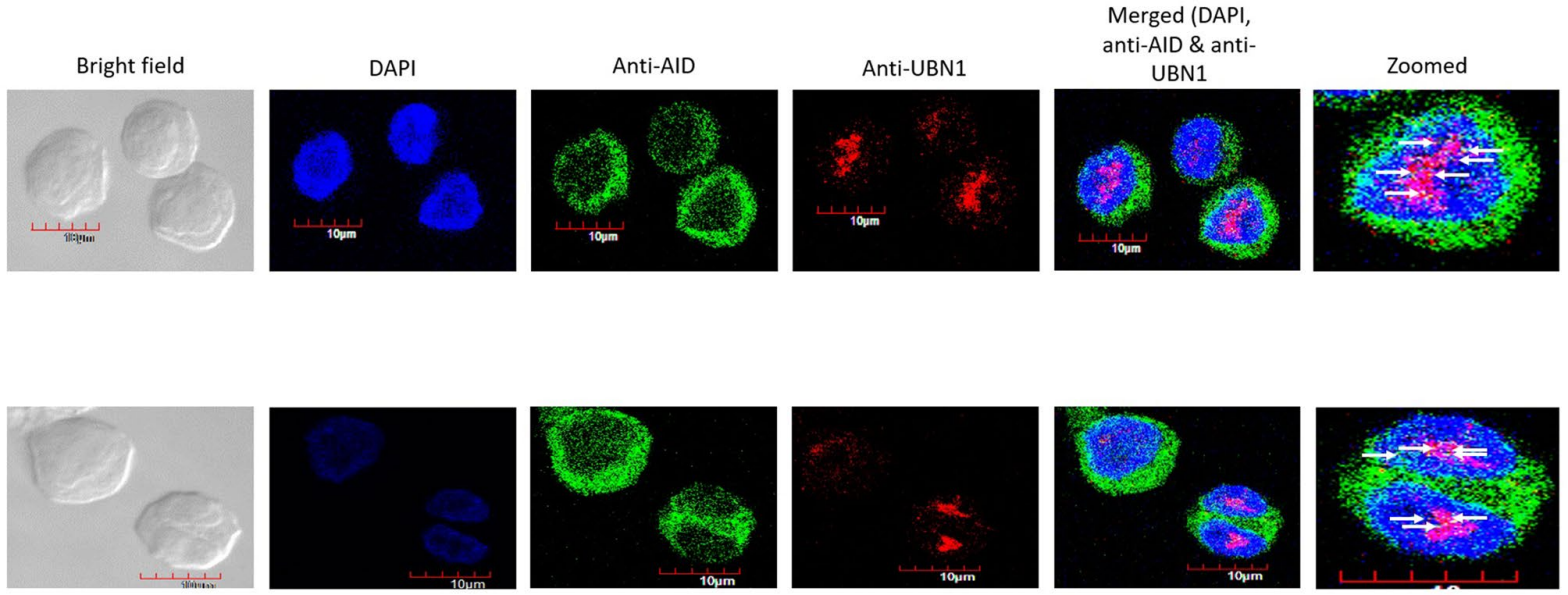
Co-localization of AID with UBN1 in DT40ѰV KO cells. Cells were fixed and double stained with monoclonal anti-AID (green) and polyclonal anti-UBN1 (red) antibodies. AID is predominantly located in the cytoplasm, whereas UBN1 is mostly inside the nucleus. Yellow and orange spots are produced due to the co-localization of AID with UBN1**.**

**Figure 8:**
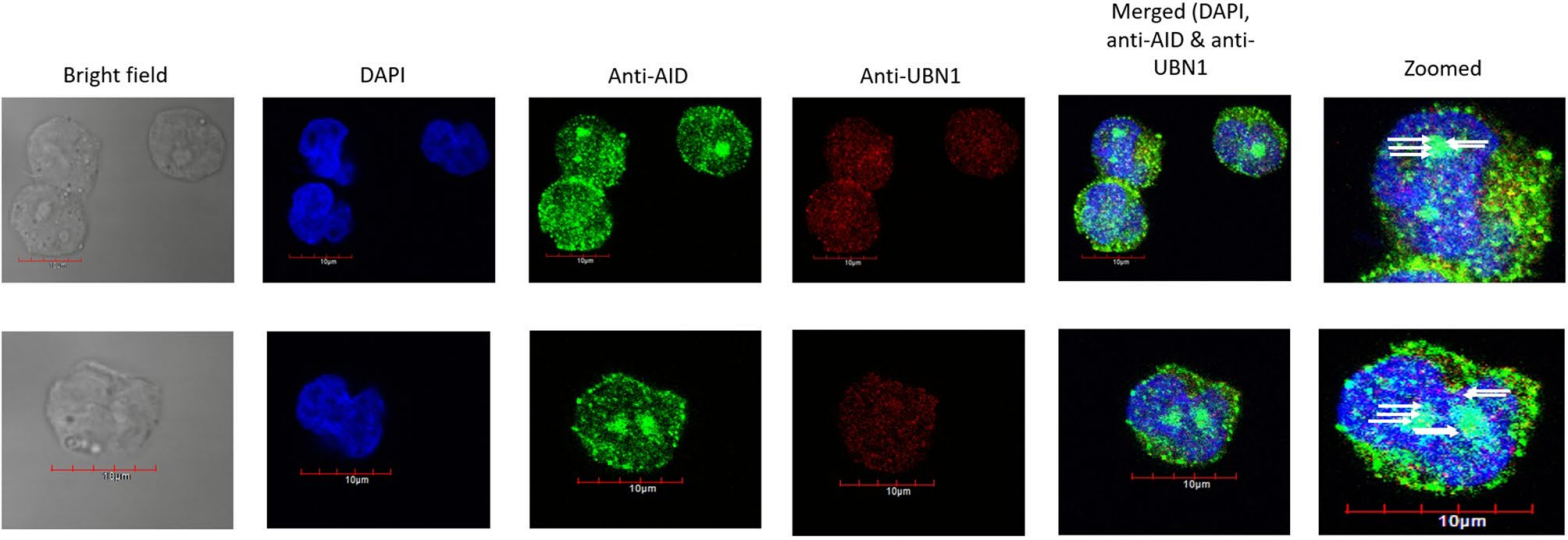
Co-localization of AID with UBN1 in Raji cells. Cells were fixed and double stained with monoclonal anti-AID (green) and polyclonal anti-UBN1 (red) antibodies. AID is predominantly located in the cytoplasm, whereas UBN1 is mostly inside the nucleus. Yellow and orange spots are produced due to the co-localization of AID with UBN1**.**

As our in-silico and co-immunoprecipitation data indicate that AID interacts with UBN1, we wanted to confirm whether UBN1 and AID are co-localized inside the B-cells. Thus, we performed a co-localization assay of both proteins using double immunofluorescence staining with anti-AID monoclonal antibody and anti-UBN1 polyclonal antibody in Raji, as well as in DT40ψV KO cells (Figs. 7 & 8, Figs. S10, S11, S12). As expected, AID is predominantly localized to the cytoplasm as it consists of a weak nuclear localization signal and a strong nuclear export signal, whereas a small fraction of AID is localized in the nucleus. On the contrary, UBN1 is primarily localized in the nucleus of DT40ψV KO cells, whereas it is distributed throughout the cytosol and nucleus in Raji cells. As UBN1 is a part of the HIRA chaperon complex that acts on chromatin, its predominant localization in the nucleus is indispensable for regulating chromatin dynamics. However, several yellow and orange spots were observed inside the nucleus of Raji and DT40ψV KO cells, which validated our previous observations in the co-immunoprecipitation assay that UBN1 interacts with AID inside B-cells. Further, the co-localization assay was also performed on DT40 AID KO cells to eliminate any non-specific co-localization of UBN1 and AID. As anticipated, due to the absence of AID protein, the co-localization assay on DT40 AID KO cells (Fig. S13) reveals no significant discrete green or yellow signal. So, it eliminates the possibility of non-specific binding of AID antibody. Thus, we can tell that signal of AID and UBN1 co-localization in DT40ѰV KO cells is due to the presence of each other at the same location and not due to a non-specific signal.

**Figure 9:**
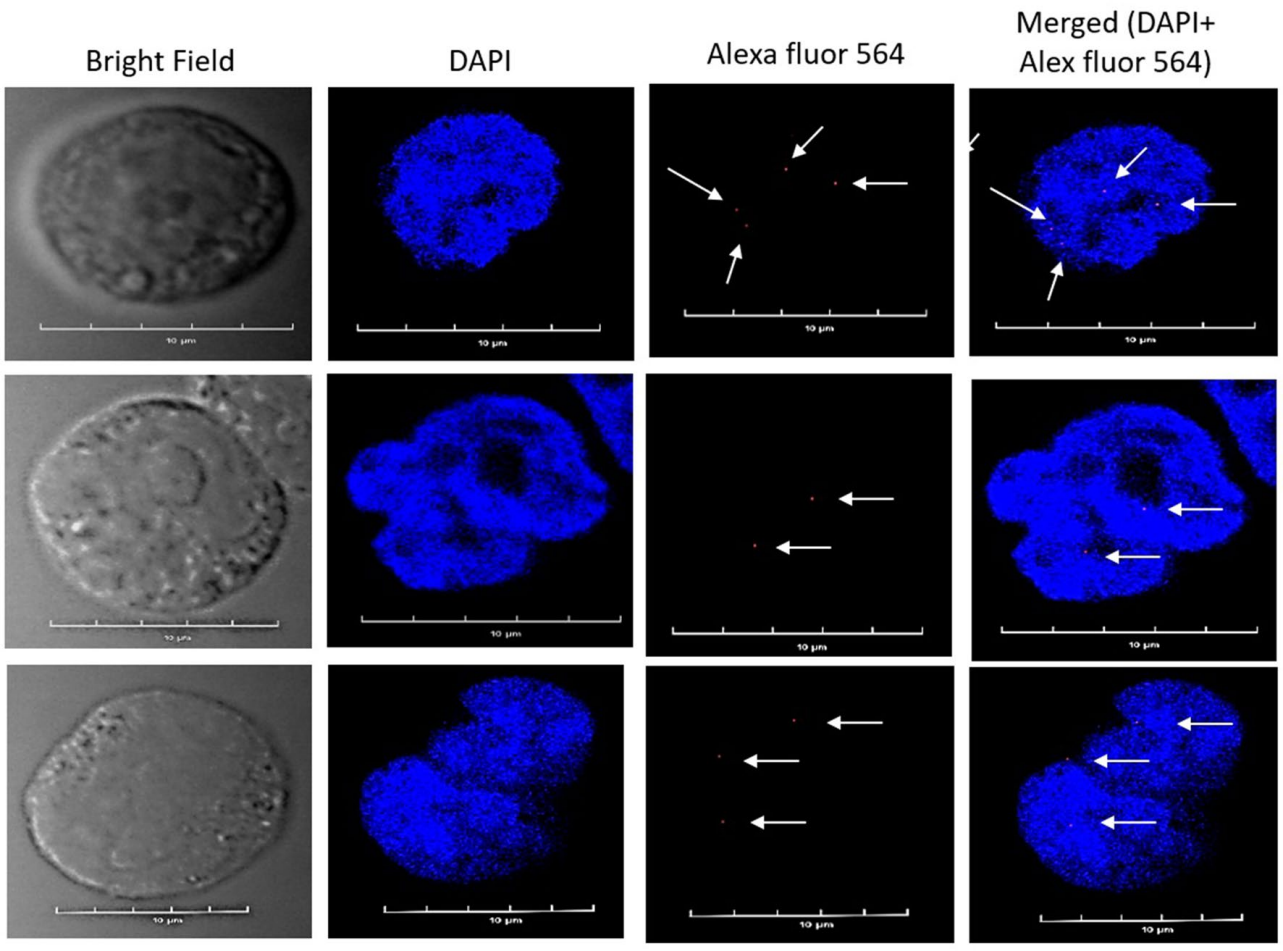
Proximity ligation assay for AID and UBN1 in Raji cells. Images demonstrate the PLA signals in Raji cells and the proximity between two proteins, AID and UBN1. Nuclei were stained with DAPI. Anti-AID and anti-UBN1 primary antibodies were incubated, followed by incubation with secondary antibodies conjugated to oligonucleotides (PLA Probes anti-mouse MINUS and PLA Probes anti-rabbit PLUS). Red spots in the cells shown by the arrow indicate the interaction between UBN1 and AID.

**Figure 10:**
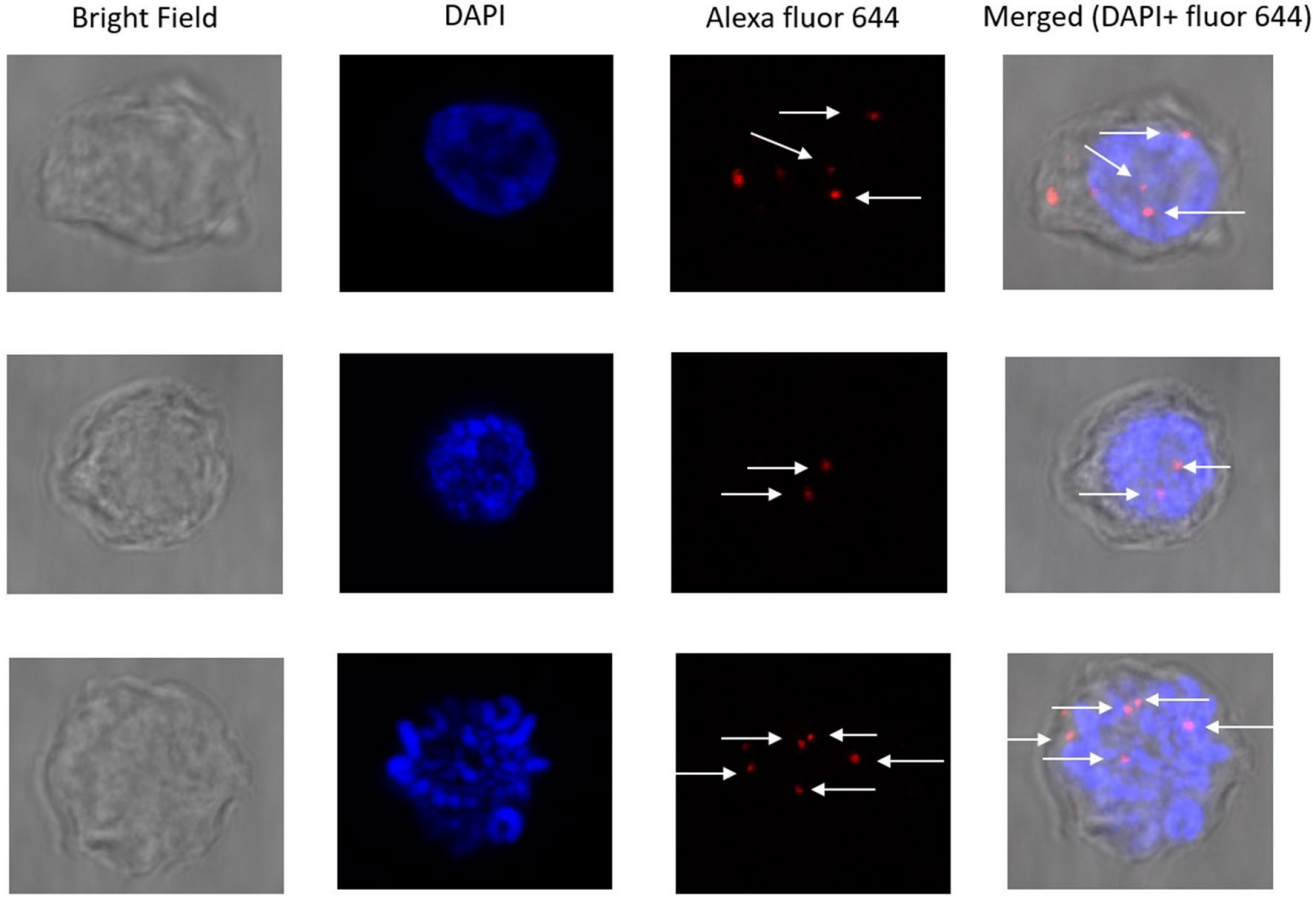
Proximity ligation assay for AID and UBN1 in DT40ψV KO cells. Images demonstrate the PLA signals in DT40ψV KO and the proximity between two proteins, AID and UBN1. Nuclei were stained with DAPI. Anti-AID and anti-UBN1 primary antibodies were incubated, followed by incubation with secondary antibodies conjugated to oligonucleotides (PLA Probes anti-mouse MINUS and PLA Probes anti-rabbit PLUS). Red spots in the cells shown by the arrow indicate the interaction between UBN1 and AID.

Additionally, to understand the molecular interaction between UBN1 and AID, we performed a proximity ligation assay. Raji cells were incubated with anti-UBN1 and anti-AID antibodies; secondary antibodies (as mentioned by the manufacturer) were subsequently added. Strikingly, we detected PLA spots (red) in Raji cells but the intensity and number of spots were quite less (Fig. 9). Furthermore, all the PLA spots were found inside the nucleus. As optimum PLA spots can only be formed if the two proteins are within the range of 10 Å, it suggested that the interaction between UBN1 and AID could be either weak or transient. Similarly, the PLA assay was also performed on DT40ѰV KO cells (Fig. 10), and we detected PLA spots that suggest UBN1 and AID interaction inside the cell. Thus, our PLA data demonstrates AID and UBN1 interaction is direct in both DT40ѰV KO cells and Raji cells.

## Discussion

SHM and CSR are two key events in germinal centre B-cells, resulting in antibody diversity. Interestingly, activated B-cells produce an enzyme known as AID that mediates SHM and CSR through the deamination of cytosine to uracil at the Ig locus, creating a single nucleotide mismatch. Further, the cell's DNA repair pathways quickly scan AID-created DNA mismatches. Surprisingly, the mismatches at the Ig locus led to the recruitment of downstream error-prone DNA polymerase that finally incurred mutations^41^. AID-created mutations at Ig genes are key for survival as it produces higher affinity antibodies and switching from one isotype to another (IgM/IgD to IgG or IgA). Purified AID protein showed an extremely high affinity for binding to the single-stranded DNA across the species irrespective of dissimilarity in the sequence^8^. Moreover, cell-free extract studies highlighted that AID could deaminate any cytosine residue, but it preferably deaminates at cytosine residue in the WRC region (also known as AID hotspots)^42^. Interestingly, AID is an inefficient enzyme as it can deaminate only a small fraction of cytosine residue in the WRC region it encounters. In B-cells, AID activity is precisely regulated at an optimal level as deficient or lower AID activity leads to hyper IgM syndrome (decreased immunity or immunocompromised)^43^. In contrast, aberrant activity of AID can drive the cells toward several types of B-cell lymphoma and carcinoma^2^.

Targeting AID to the Ig genes requires single-stranded DNA, which is why AID is linked with transcription^10^. Furthermore, transcription initiation^10^ and RNA polymerase pausing are crucial for targeting AID to the Ig locus^11^. During transcription, AID accessibility to single-stranded DNA is still affected by the nucleosome structure and positioning sequence^44, 45^. Interestingly, to overcome the nucleosome barrier and make DNA more accessible to AID at Ig genes for productive SHM or CSR, B-cells deploy different chromatin modifiers. In fact, the Ig locus in B-cells is highly enriched with the FACT complex^12^ and the HIRA chaperon complex, which later paves a path for the deposition of non-canonical histone H3.3^13^. Moreover, it is well documented that H3.3 deposition at the Ig locus is the hallmark of SHM^12^ as it provides the AID-accessible single-stranded DNA^46^. Further, a study using DT40 cells depleted with H3.3 showed diminished SHM without affecting the level of transcription in contrast to wild-type cells. Thus, H3.3 plays a pivotal role in AID-mediated SHM^46^. Even before the discovery of the H3.3 role in SHM, it is well established that HIRA plays a crucial role in the deposition of H3.3 at transcriptionally active genes^46, 47^. Additionally, UBN1 (a member of the HIRA chaperon) binds to HIRA through its NHRD domain that interacts with the WD40 domain of HIRA^48^. Interestingly, the UBN1 HRD domain specifically interacts with H3.3/H4 and is responsible for de novo deposition of H3.3/H4 to the DNA^49^. A recent study unfolds the role of the HIRA complex in SHM. In Ramos cells, they demonstrated that HIRA KO significantly impairs the somatic mutation at the Ig locus^13^. UBN1 plays a crucial role in HIRA/ASF1a in the chromatin remodeling pathway in senescent cells^48^.

**Figure 11:**
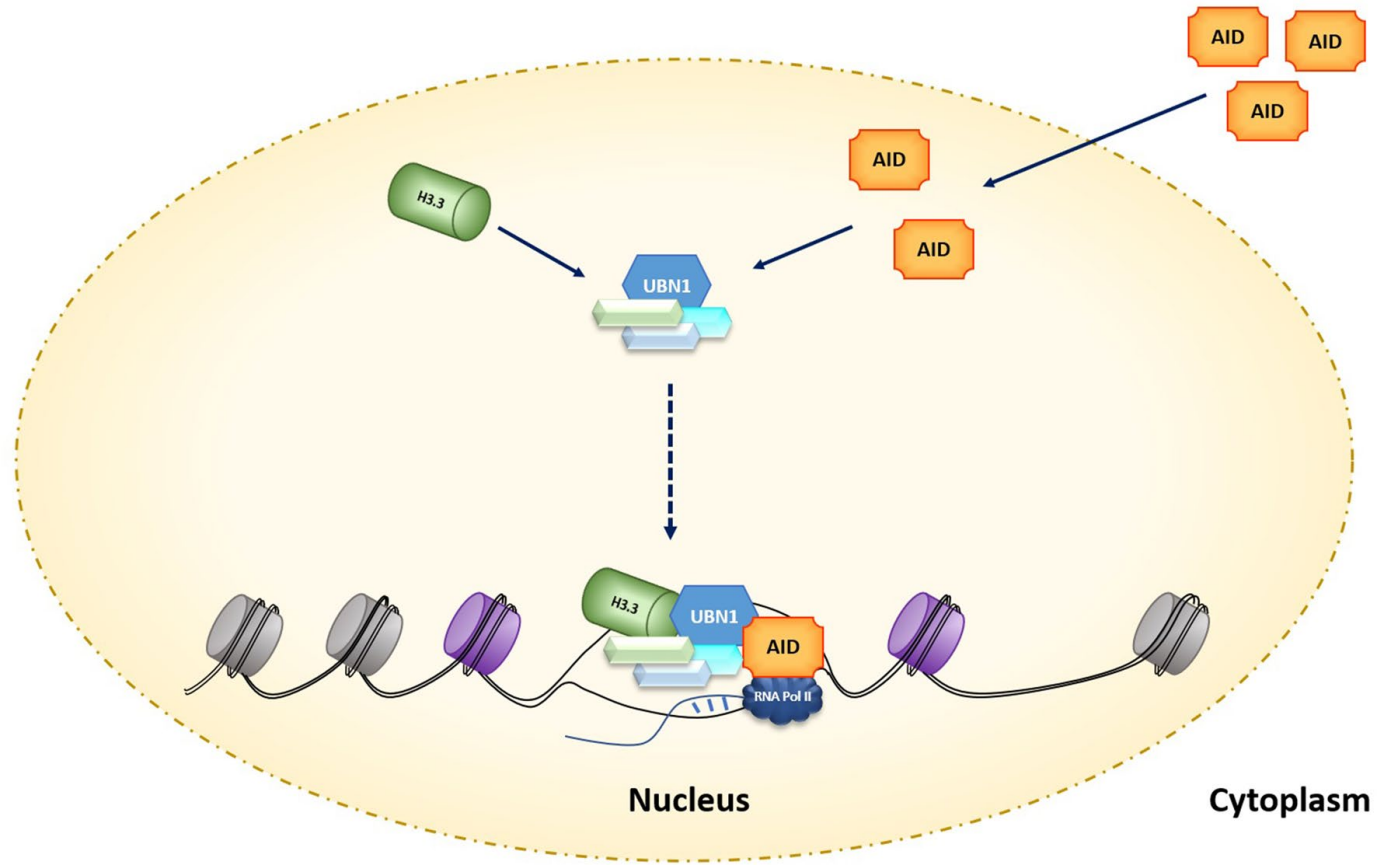
UBN1 interacts with AID as well as H3.3. Due to this interaction, UBN1 may be able to load H3.3 and AID in complex forms to the variable region of the Ig locus.

Our molecular docking and MD simulation study showed that AID interacts with UBN1. Additionally, co-immunoprecipitation assays from cell lysate of chicken and human cell lines confirm AID-UBN1 interactions (Figs. 3 & 4). Further, in vitro pull-down assay with cell lysate (Fig. 5) as well as with purified proteins (Fig. 6) also validates the interaction of UBN1 and AID as deduced via co-immunoprecipitation. Moreover, co-localization using fluorescently labelled antibodies showed that AID and UBN1 co-localized in the cells. Interestingly, the PLA assay also suggests that interaction between UBN1 and AID is direct but may be either weak or transient. Additionally, the PLA spots were also seen in the DT40ψV KO chicken cells (Fig. 10). As UBN1 (is a part of the HIRA chaperon complex) and is also found to interact with H3.3, UBN1, AID, and H3.3 may be a part of the same complex, which gets deposited at the Ig locus and assist in AID mediated SHM (Fig. 11). Interestingly, RNA seq data documented in the literature clearly showed that HIRA, UBN1, and H3.3 are upregulated in germinal centre B-cells compared to naive B-cells^49, 50^. Moreover, overexpressed UBN1 in collaboration with HIRA and H3.3 and its interaction with AID may have a role in SHM of Ig genes still needs to be elucidated. Recently, the direct significance of HIRA protein was unfolded in SHM^13^. The HIRA KO Ramos cells showed a significant decrease in SHM without affecting the expression of SHM-specific protein. Furthermore, ChIP data suggest a steep decline in the occupancy of H3.3 at the Ig locus in HIRA KO cells in contrast to the wild type. Clearly, HIRA-mediated H3.3 deposition at the IgV locus is crucial for activated B-cells undergoing hypermutation^13^. Surprisingly, HIRA KO cells shifted AID-mediated mutation from AID hotspot to non-hotspot regions. Thus, it is quite possible that UBN1-AID interaction restricts the AID activity to the AID hotspot and prevents random mutation to the non-hotspot areas at the Ig locus. Recently, SRSF1-3 (required for SHM in DT40 cells) reconstitute cells also showed a higher level of UBN1^23^. Incidentally, a higher level of UBN1 results in its increased occupancy at the Ig locus, and H3.3 was also enriched at the Ig locus^23^. AID-mediated SHM is a complex process involving many factors that directly and indirectly affect it. Nevertheless, the fate of UBN1 and AID interaction in the context of SHM needs to be explored and elucidated.

### Supplementary Information

Supplementary Information.

## Data Availability

The raw data are provided by the corresponding author upon reasonable request.
