# Supplementary material for: Activation-induced cytidine deaminase an antibody diversification enzyme interacts with chromatin modifier UBN1 in B-cells
Source: Sci Rep. 2023 Nov 10;13:19615. doi: 10.1038/s41598-023-46448-7 (PMC10638239; doi:10.1038/s41598-023-46448-7)
Supplement: Supplementary file 1 — Supplementary Information. [file 41598_2023_46448_MOESM1_ESM.pdf]

Supplementary Information for

Activation-induced cytidine deaminase an antibody diversification enzyme interacts with chromatin modifier UBN1 in B-cells

Ankit Jaiswal[1], Rajarshi Roy[1], Anubhav Tamarkar[1], Amit Kumar Singh[1], Parimal Kar[1] and Prashant Kodgire[1]*

[1]Department of Biosciences and Biomedical Engineering, Indian Institute of Technology Indore, Indore - 453 552, Madhya Pradesh, India

* Correspondence should be addressed to Dr. Prashant Kodgire

Email: pkodgire@iiti.ac.in

Phone: +91-731 660 3355

	ΔE_{vdW}	ΔE_{elec}	ΔG_{pol}	ΔG_{np}	ΔE_{MM}^{a}	ΔG_{solv}^{b}	ΔG_{bind}^{c}
UBN1-Aid complex	-29.90 (0.07)	-167.05 (0.59)	169.88 (0.54)	-4.13 (0.01)	-196.94 (0.58)	165.76 (0.54)	-31.18 (0.11)

a $=\Delta E_{vdW +}\ \Delta E_{elec}$

b $=\Delta G_{pol+}\ \Delta G_{np}$

c $=\Delta E_{vdW +}\ \Delta E_{elec} + \Delta G_{pol+}\ \Delta G_{np}$

Table. S1. Binding free energy components (kcal/mol) for the binding of UBN1-AID complex. $\mathbf{\Delta E_{vdW}}$**:** van der Waals energy; $\mathbf{\Delta E_{elec}}$**:** electrostatics energy in the gas phase; $\mathbf{\Delta G_{pol}}$**:** polar solvation energy; $\mathbf{\Delta G_{np}}$**:** nonpolar solvation energy; $\Delta \mathbf{G_{bind}}$**:** total binding free energy.

Cloning and expression of various proteins

UBN1$^{1-175}$ and hAID were PCR amplified from the cDNA of Raji cells using primers PK 934 & PK 935 and PK 774 & PK 910 (**Table S2**) and cloned at *Bam*HI/*Hind*III in His-MBP vector to create His-MBP-Flag-UBN11-175 and His-MBP-hAID, respectively. Similarly, to create clones without His-tag, we removed His-MBP from His-MBP-Flag-UBN1 and His-MBP-hAID by restriction digestion with *Nde*I and *Nhe*I, and cloned only MBP (amplified using primers PK 957 and PK 960, **Table S2**) at *NdeI*/*Nhe*I site to give rise to MBP-Flag-UBN1$^{1-175}$ and MBP-hAID without His-tag, respectively. Subsequently, each protein was expressed in the Rosetta strain of *E. coli.* Proteins were induced by 0.5 mM IPTG at kept at 16 °C/18 hrs/180 rpm. His-tagged proteins were purified either on the HisTrap (Cytiva) column, whereas, proteins without His-tag were purified on the MBP-trap (Cytiva) column, according to the manufacturer's protocol. An empty vector was used to express His-MBP only.

Table S2 List of primers used in the study**.**

Primer Name	Primer Sequence
PK957	5'-AAAACATATG AAA ATA AAA ACA GGT GCA CGC ATC-3'
PK960	5'-AAAAGCTAGCCTTGGTGATACGAGTCTGCG-3'
PK774	5'-GCTTGGATCCGACAGCCTCTTGATGAACCGGAG-3'
PK910	5'- CGTAAGCTTTCAAAGTCCCAAAGTACGAAATG-3'
PK934	5'-GCTTGGATCCGACTACAAAGACGATGACGACAAGTCGGAGCCCCACAGGGTC-3'
PK935	5'-CGT AAGCTT TCAAGACTCTGATGCTTGTCTAAACTGCAGGG-3'

Fig. S1

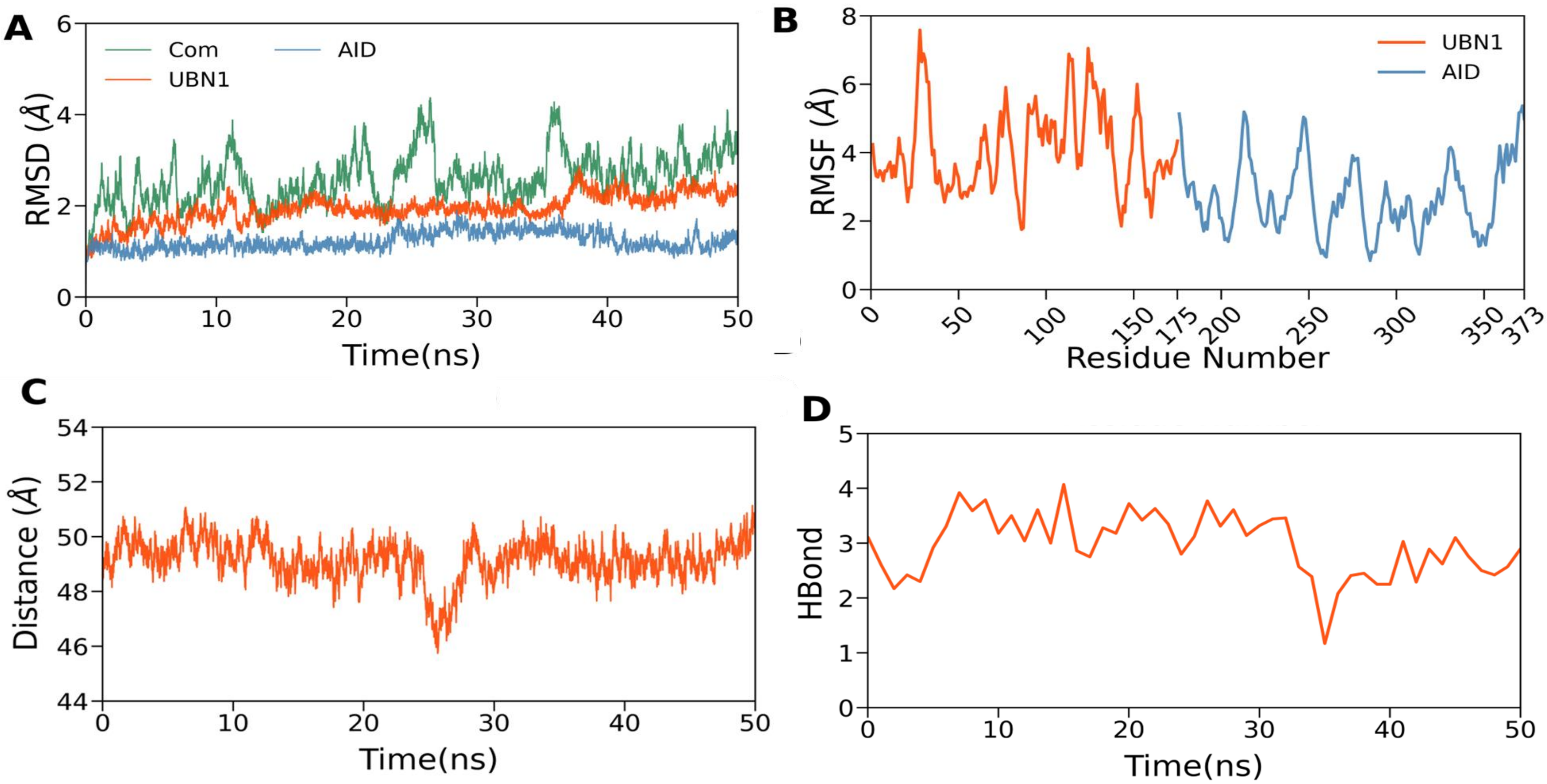

Fig. S1. (**A**) Time evolution of root mean squared deviation (RMSD) of complex and its components, (**B**) Root mean squared fluctuation (RMSF) of UBN1 and AID, (**C**) Time evolution of protein-protein center of mass distance, (**D**) Time evolution of hydrogen bonds between UBN1 and AID.

Fig. S2

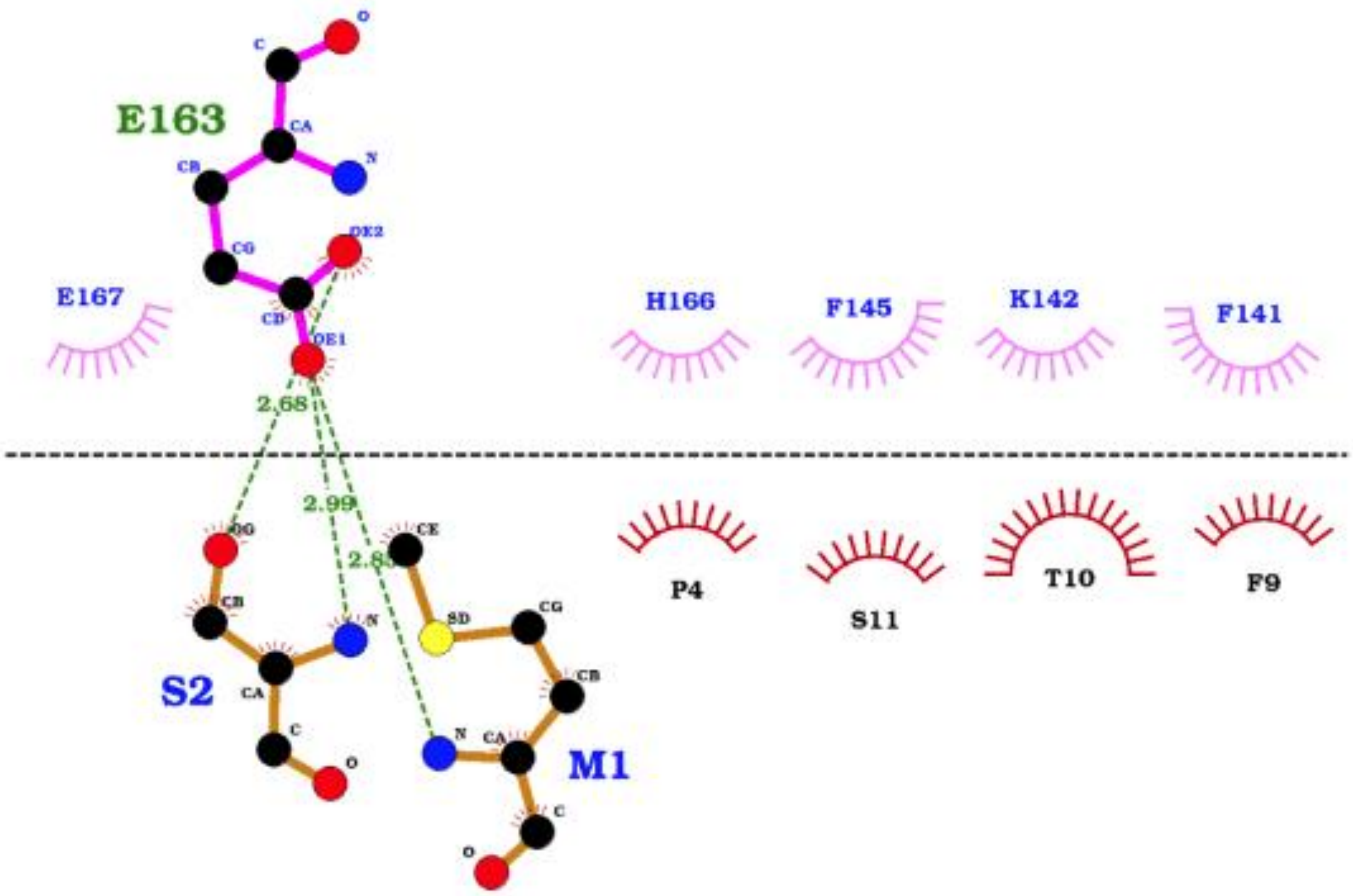

Fig. S2. The protein-protein interaction profile between UBN1 and AID was constructed using Ligplot⁺. Hydrogen bonds are shown in the green dotted line. Semicircles show the residues involving hydrophobic contacts.

Fig. S3

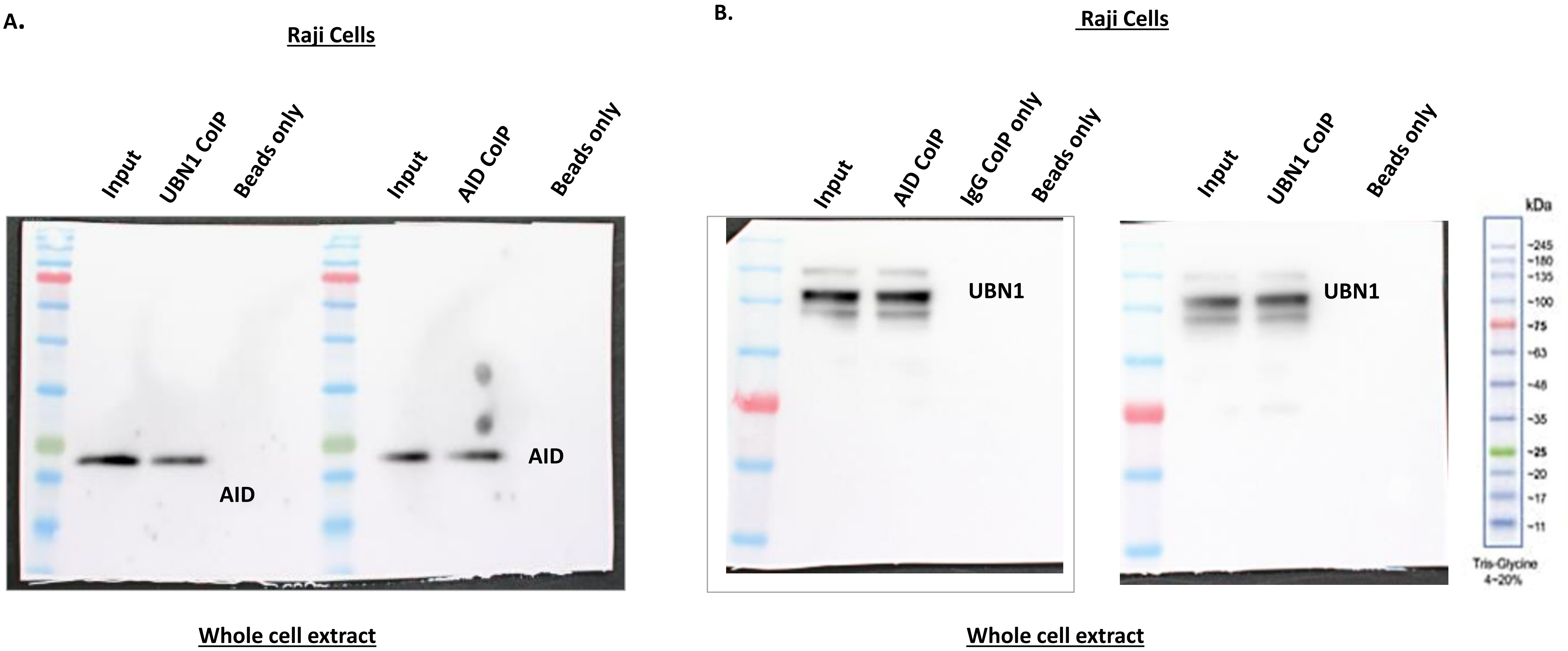

Fig. S3. The co-immunoprecipitation original image used to create **Fig. 3.** AID interaction with UBN1 in Raji cells. **(A)** Co-immunoprecepitation of UBN1 from whole cell lysate was performed from Raji cells using anti-UBN1 pAb and analyzed by western blotting using protein anti-AID mAb, AID CoIP was performed and AID was detected using anti-AID Ab. and anti-UBN1. **(B)** Co-immunoprecipitation of AID from whole cell lysate of Raji cells using anti-AID mAb and analyzed by western blotting using anti-UBN1 pAB , UBN1 was detected in UBN1 CoIP.

Fig. S4

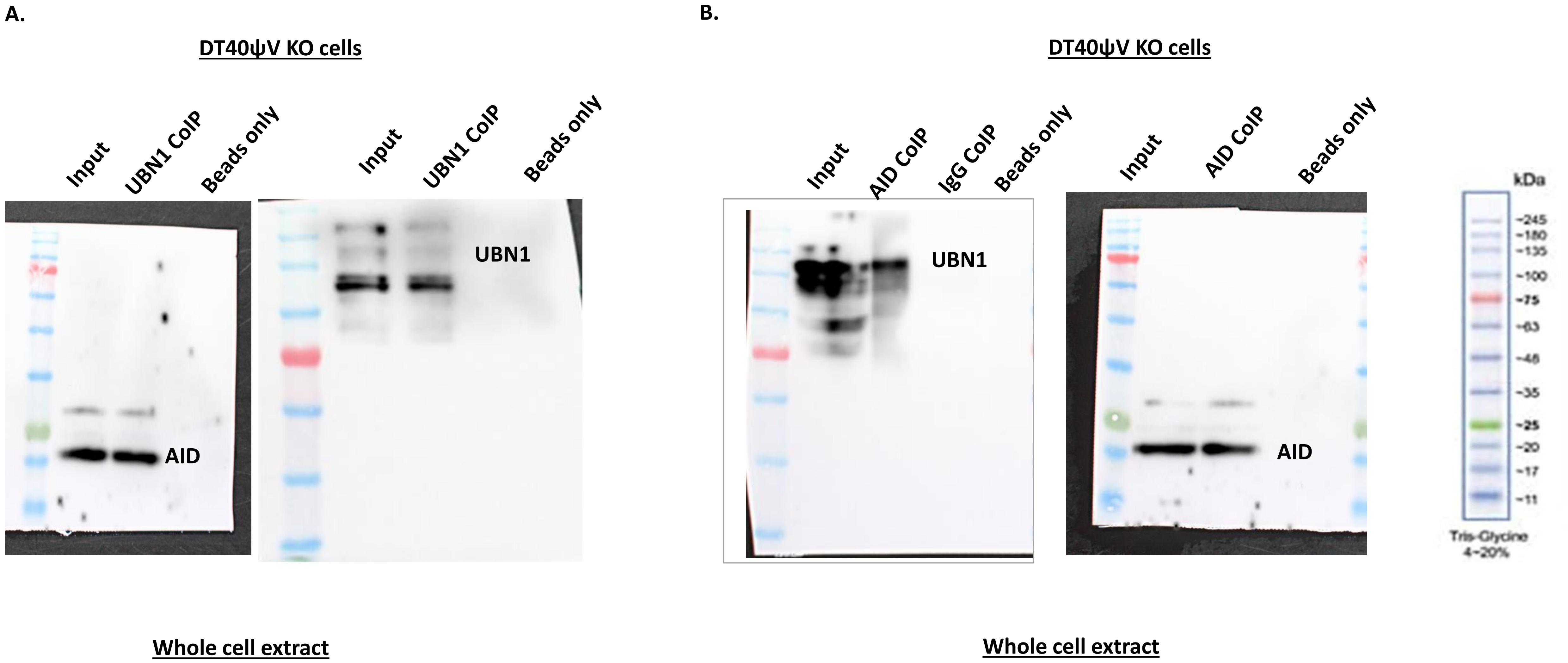

Fig. S4. The co-immunoprecipitation original image used to create **Fig. 4.** AID interaction with UBN1 in DT40ψV KO cells. **(A)** Co-immunoprecepitation of UBN1 from whole cell lysate was performed from DT40ψV KO using anti-UBN1 pAb and analyzed by western blotting using protein anti-AID mAb and anti-UBN1 pAb. **(B)** Co-immunoprecepitation of AID from whole cell lysate of DT40ψV KO cells using anti-AID mAb and analyzed by western blotting using anti-UBN1 pAB and anti-AID mAb.

Fig. S5

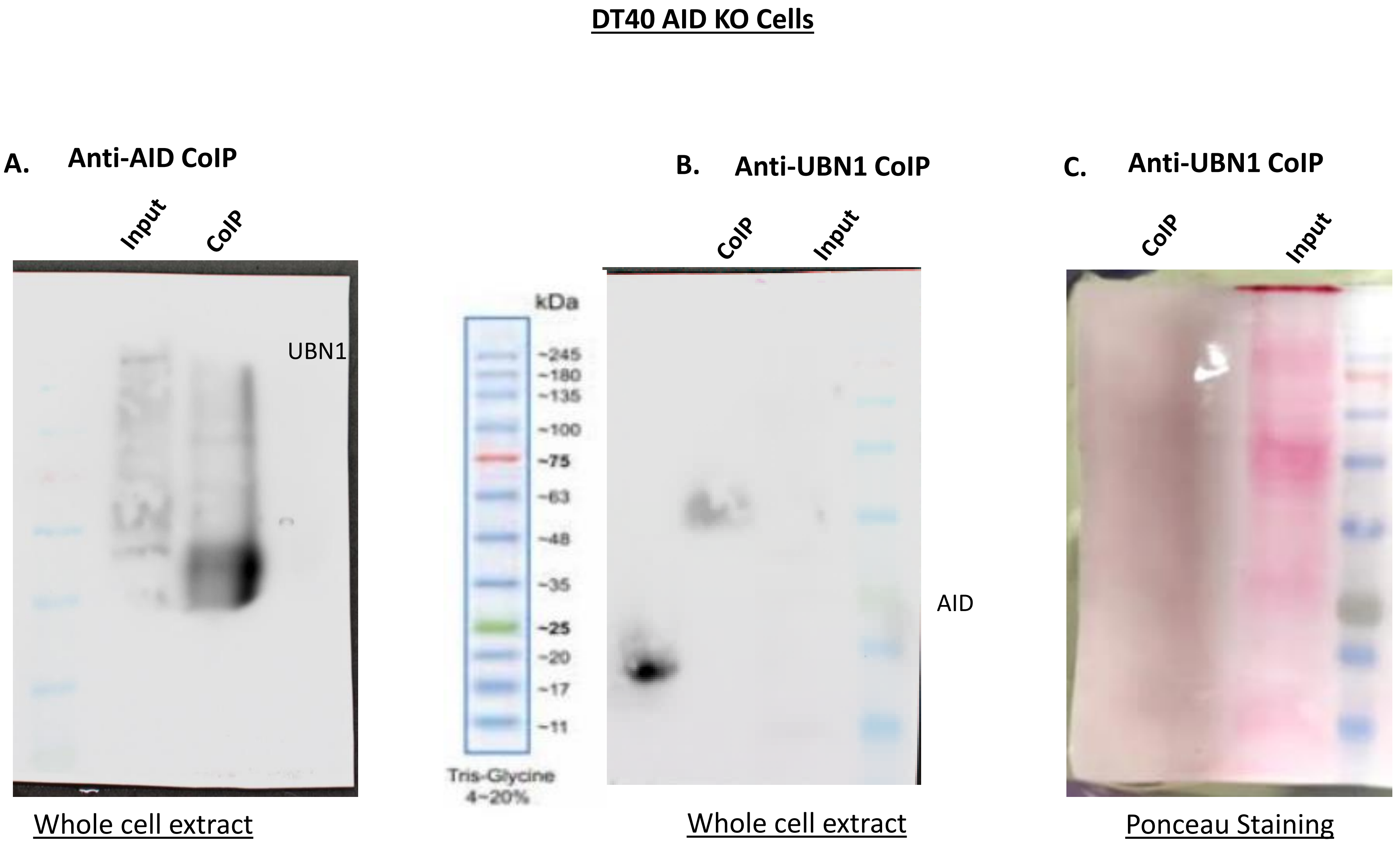

Fig. S5. Co-immunoprecepitation of AID andUBN1 in DT40 AID KO cells. **(A)** Co-immunoprecepitation of AID from whole cell lysate was performed on DT40 AID KO cells using anti-AID Ab and protein A/G beads, detection via WB using anti-UBN1 Ab. **(B)** Co-immunoprecepitation of UBN1 from whole cell lysate was performed on DT40 AID KO cells using anti-UBN1 Ab and protein A/G beads, detection vai WB using anti-AID Ab. **(C)** Blot in Fig. S5B. was stained with ponceau before adding Ab.

Fig. S6

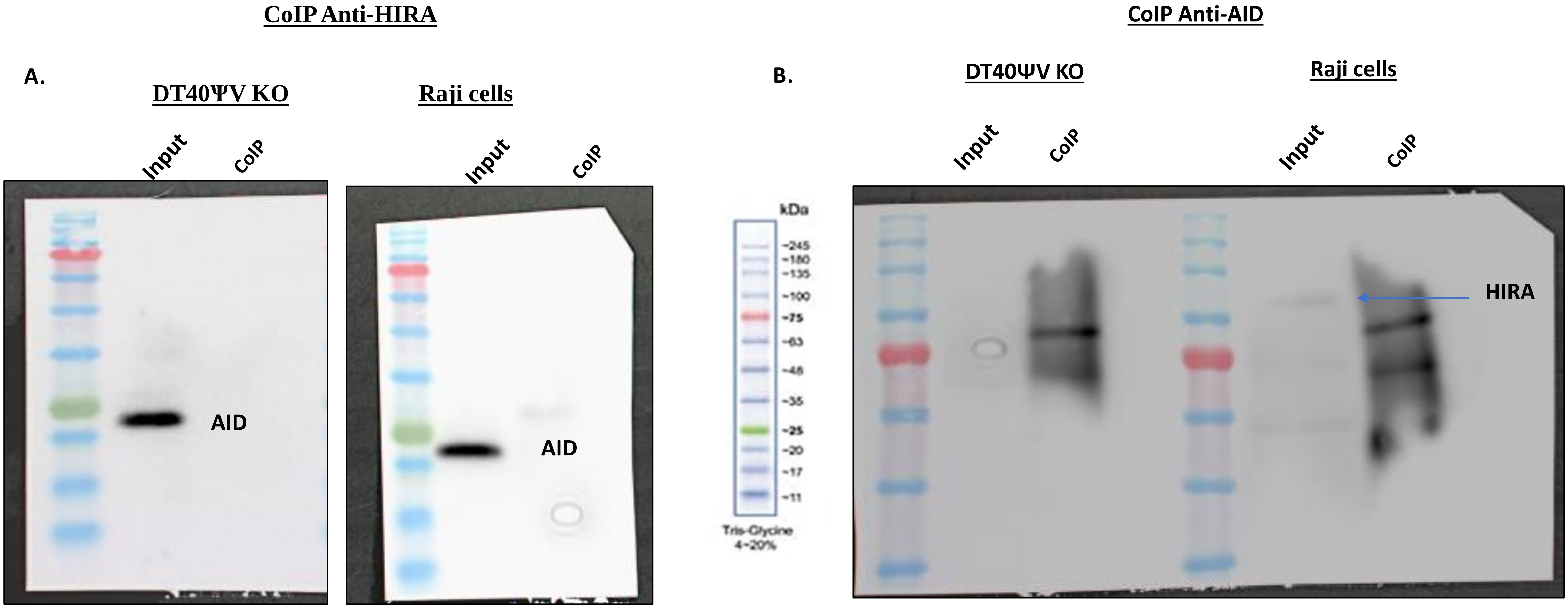

Fig. S6. AID interaction with HIRA in DT40ΨV KO & Raji cell lines. **(A)** Co-immunoprecipitation of HIRA from whole cell lysate was performed of DT40ΨV KO & Raji cell lines using anti-HIRA Ab and protein A/G beads, detection via WB using anti-AID Ab. **(B)** Co-immunoprecipitation of AID from whole cell lysate was performed of DT40ΨV KO & Raji cell lines using anti-AID Ab and protein A/G beads, detection via WB using anti-HIRA Ab

Fig. S7

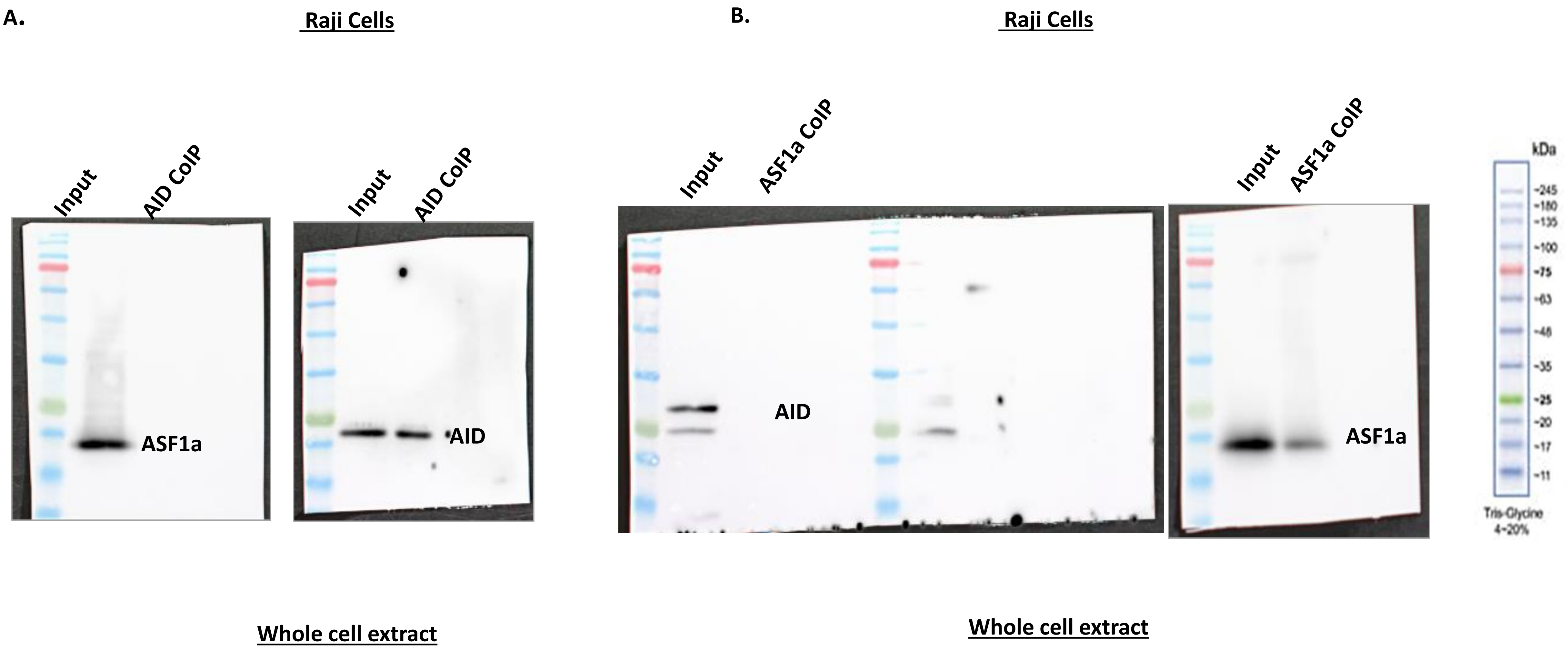

Fig. S7. AID interaction with ASF1a in Raji cells. **(A)** Co-immunoprecipitation of AID from whole cell lysate was performed from Raji cells using anti-AID Ab and analyzed by western blotting using anti-ASF1a Ab and anti-AID Ab. **(B)** Co-immunoprecipitation of ASF1a from whole cell lysate of Raji cells using anti-ASF1a Ab and analyzed by western blotting using anti-AID Ab and anti-ASF1a Ab.

Fig. S8

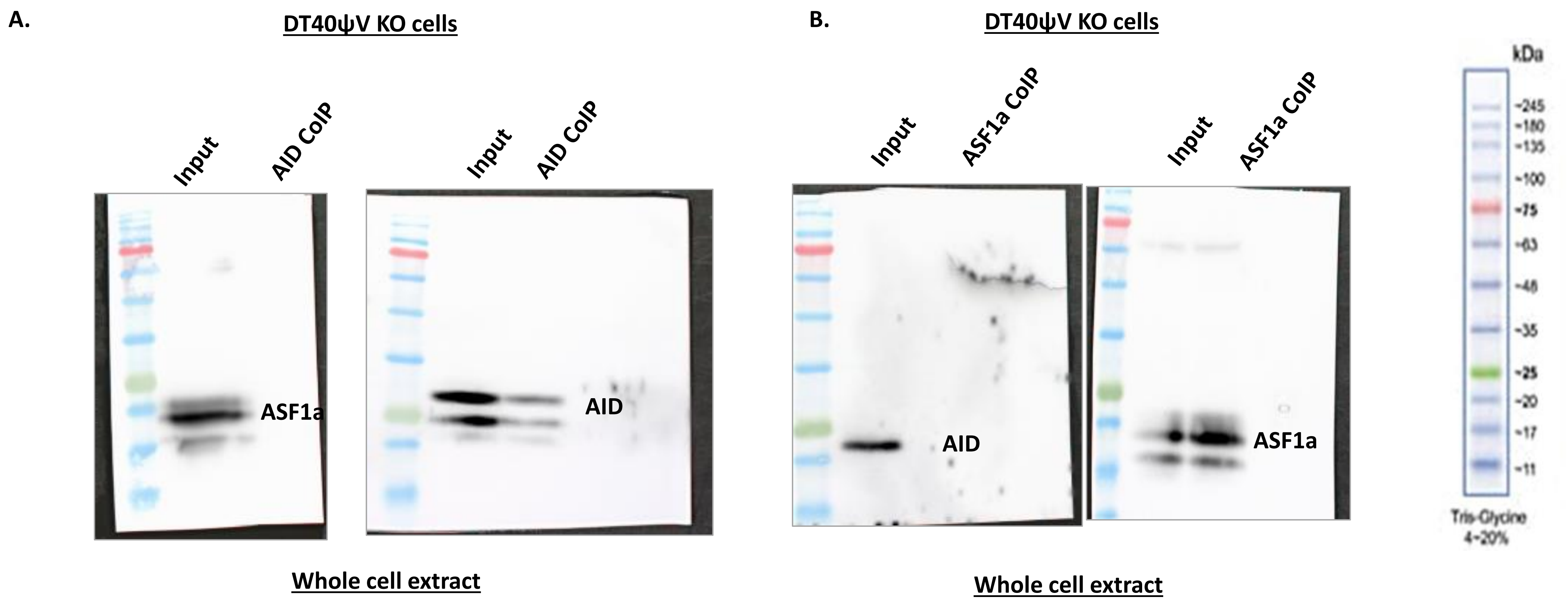

Fig. S8. AID interaction with ASF1a in DT40ψV KO cells. **(A)** Co-immunoprecipitation of AID from whole cell lysate of DT40ψV KO cells using anti-AID Ab and analyzed by western blotting using anti-ASF1a Ab and anti-AID Ab. **(B)** Co-immunoprecipitation of ASF1a from whole cell lysate of DT40ψV KO cells using anti-ASF1a Ab and analyzed by western blotting using anti-AID Ab and anti-ASF1a Ab.

Fig. S9

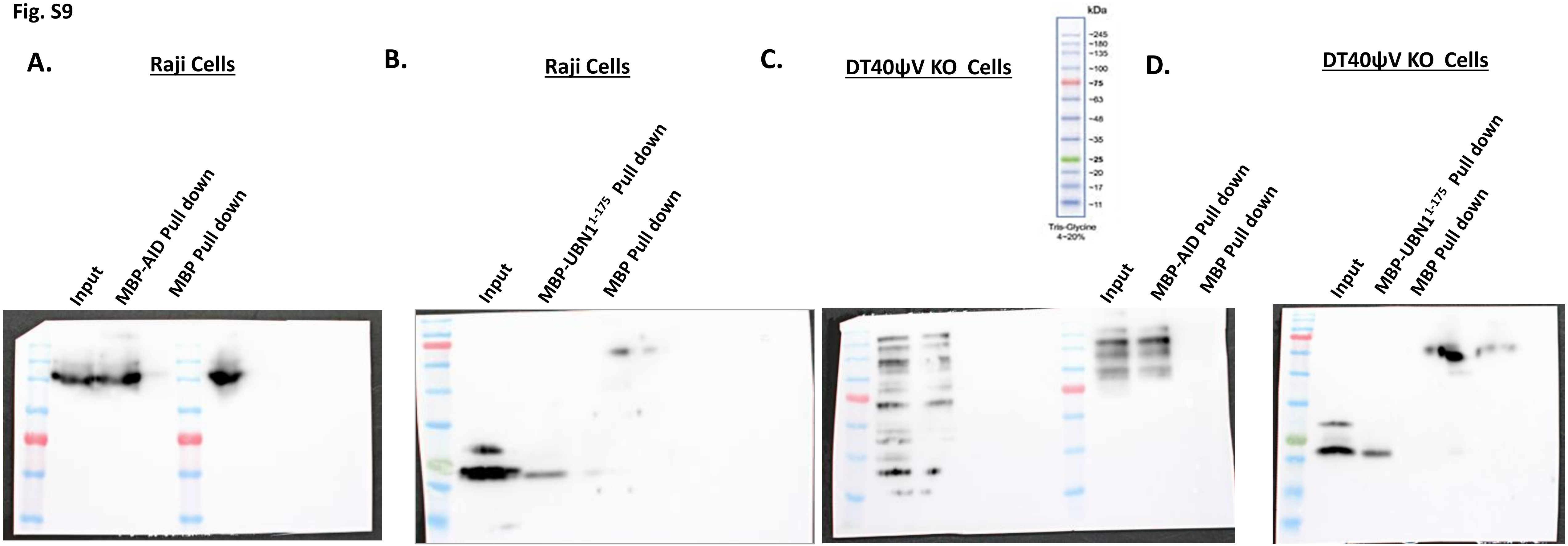

Fig. S9. The pull-down original image used to create Fig. 5 His-tagged pull-down assay for AID interaction with UBN1 in Raji cells and DT40ψV KO cells. **(A)** His tagged MBP-AID or MBP were immobilized on Ni-NTA beads followed by incubation with Raji cell lysate and analyzed by western blotting using anti-UBN1 pAb. **(B)** His tagged MBP-UBN1$^{1-175}$ or MBP were immobilized on Ni-NTA beads followed by incubation with Raji cell lysate and analyzed by western blotting using anti-AID mAb. **(C)** His tagged MBP-AID or MBP were immobilized on Ni-NTA beads followed by incubation with DT40ψV KO cell lysate and analyzed by western blotting using anti-UBN1 pAB. **(D)** His-tagged MBP-UBN1$^{1-175}$ or MBP were immobilized on Ni-NTA beads, followed by incubation with DT40ψV KO cell lysate and analyzed by western blotting using anti-AID mAB.

Fig. S10

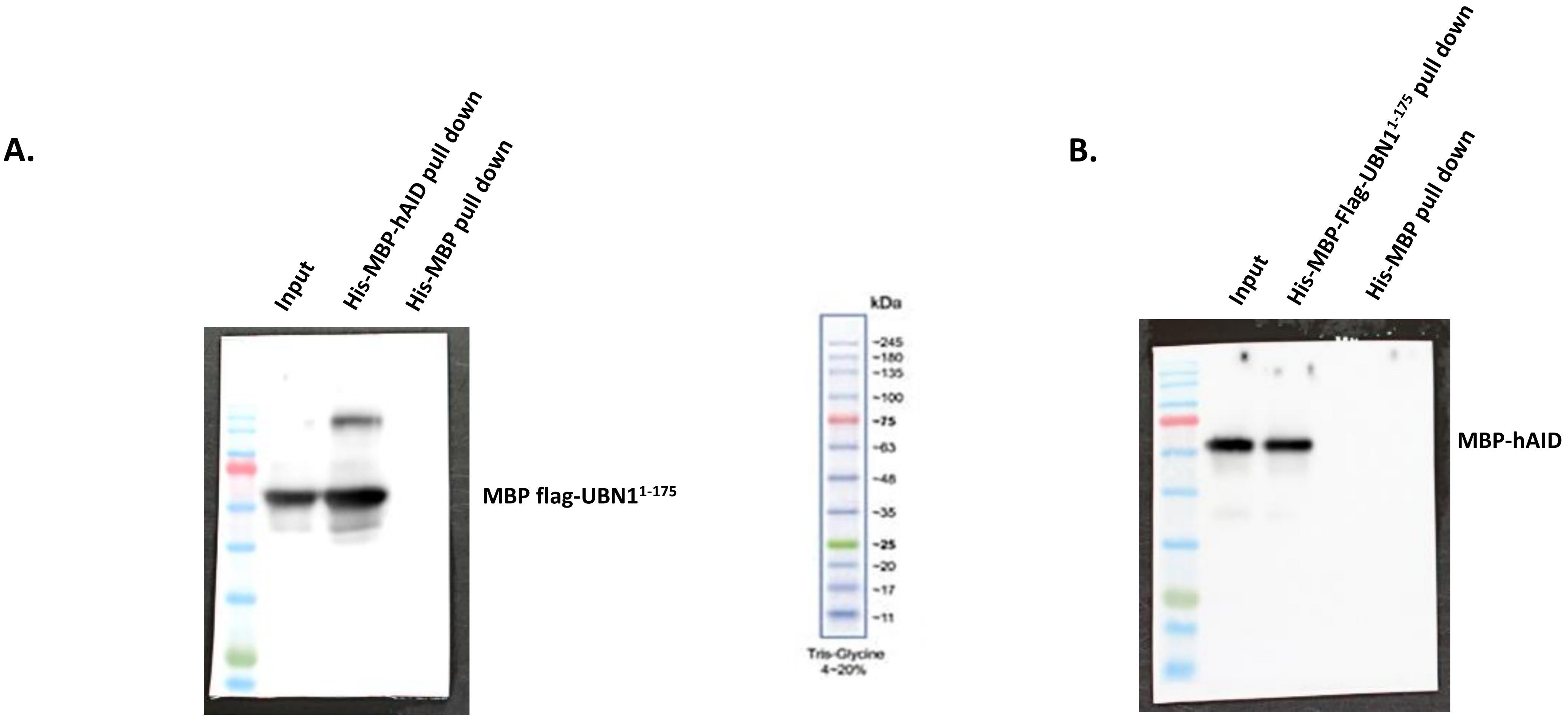

Fig. S10. Figure used to recreate main figure 6. In-vitro His-tagged pull-down assay using purified protein to detect the interaction between UBN1 and hAID. **(A)** MBP-Flag-UBN1$^{1-175}$ (w/o His tag) was added to His-MBP-hAID and His-MBP, followed by pull-down using Ni-NTA beads and detection by western blotting using anti-flag-mAb. **(B)** MBP-hAID (w/o His tag) was added to His-MBP-Flag-UBN1$^{1-175}$ & His-MBP followed by pull-down using Ni-NTA beads and detected by western blotting using anti-AID mAb.

Fig. S11

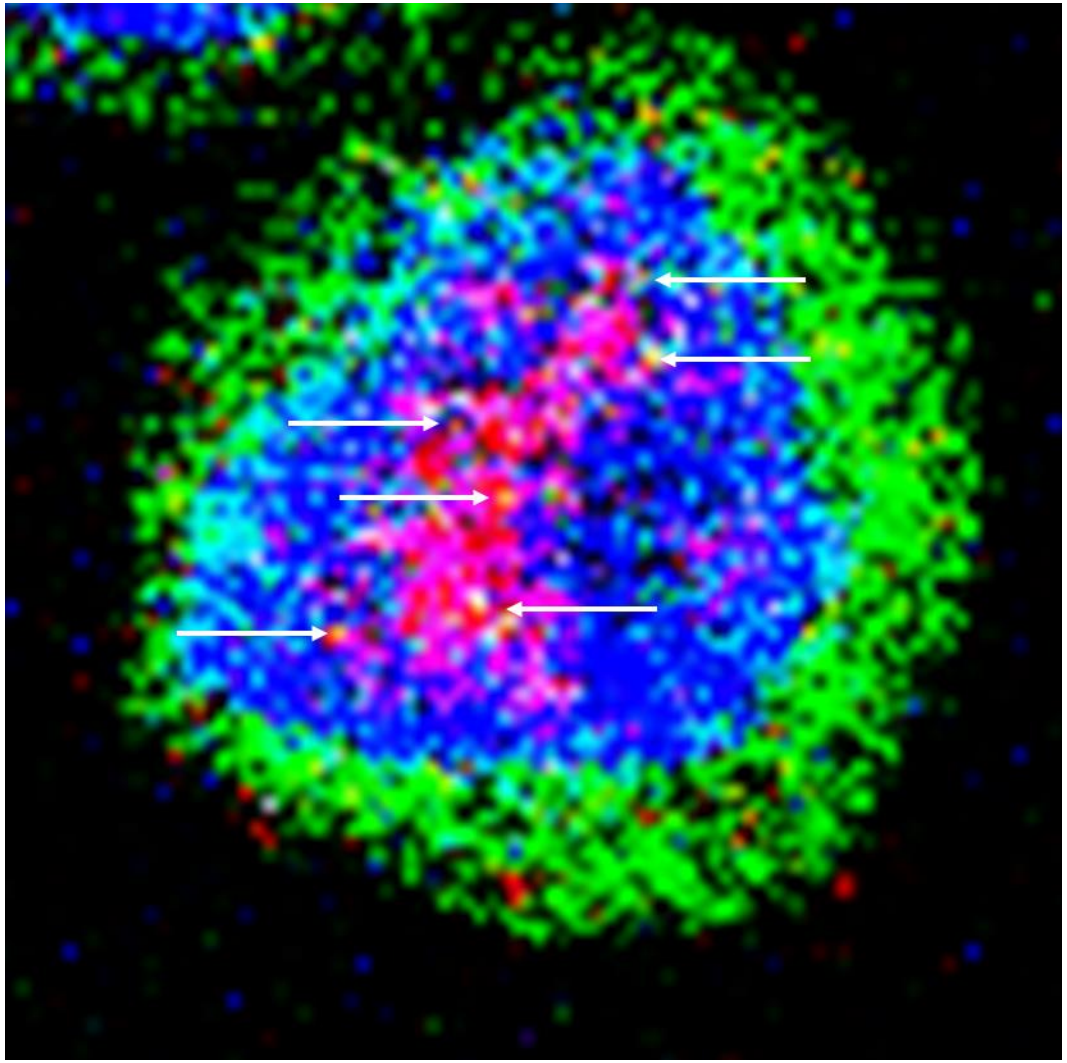

Fig. S11. Zoomed image of co-localization of UBN1 and AID in DT40ψV KO cells. Cells were stained with polyclonal anti-UBN1 (red stained) and monoclonal anti-AID (green stained). Yellow signals that merge with UBN1 and AID co-localized are highlighted with arrows.

Fig. S12

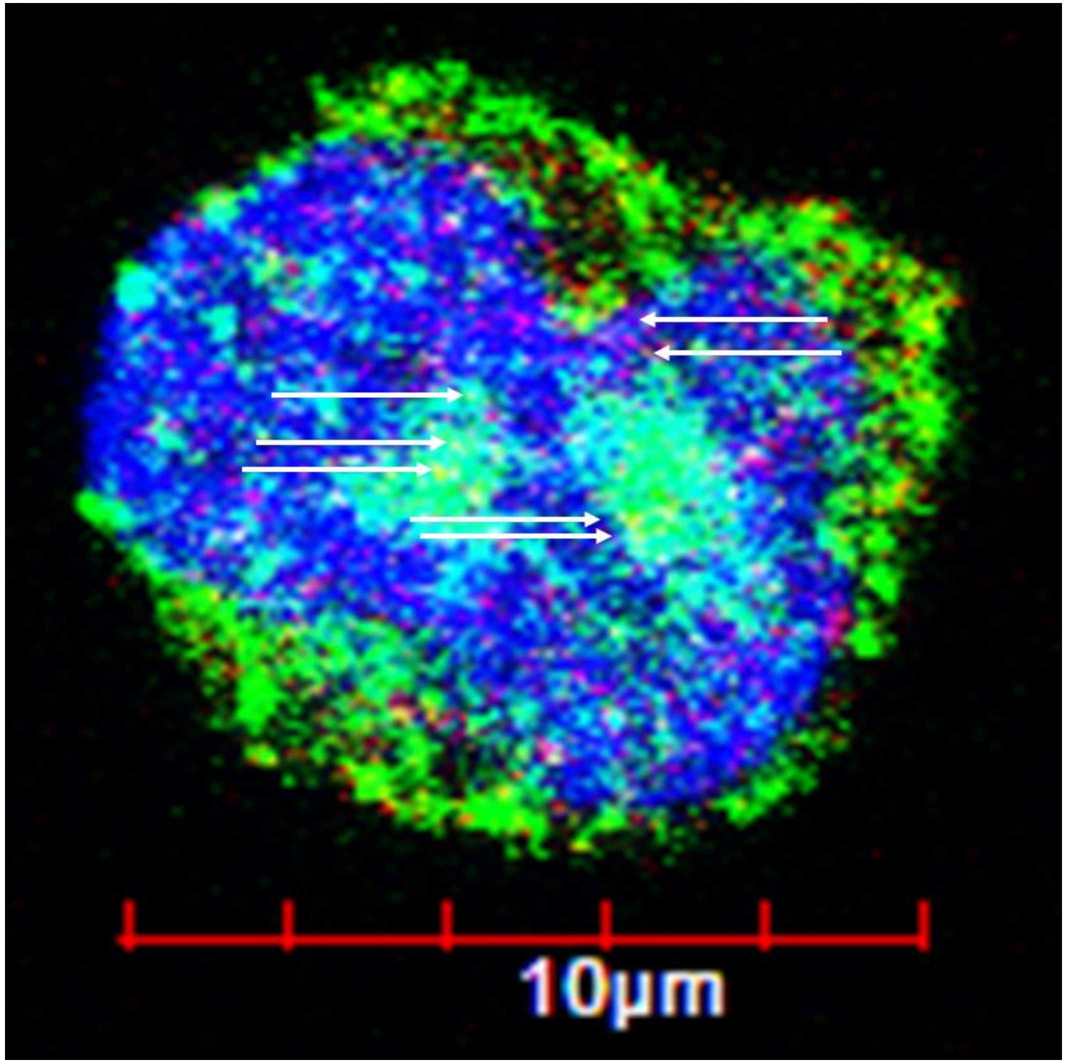

Fig. S12. Zoomed image of co-localization of UBN1 and AID in Raji cells. Cells were stained with polyclonal anti-UBN1 (red-stained) and monoclonal anti-AID (green stained). Yellow signals that merge with UBN1 and AID co-localized are highlighted with arrows.

Fig. S13

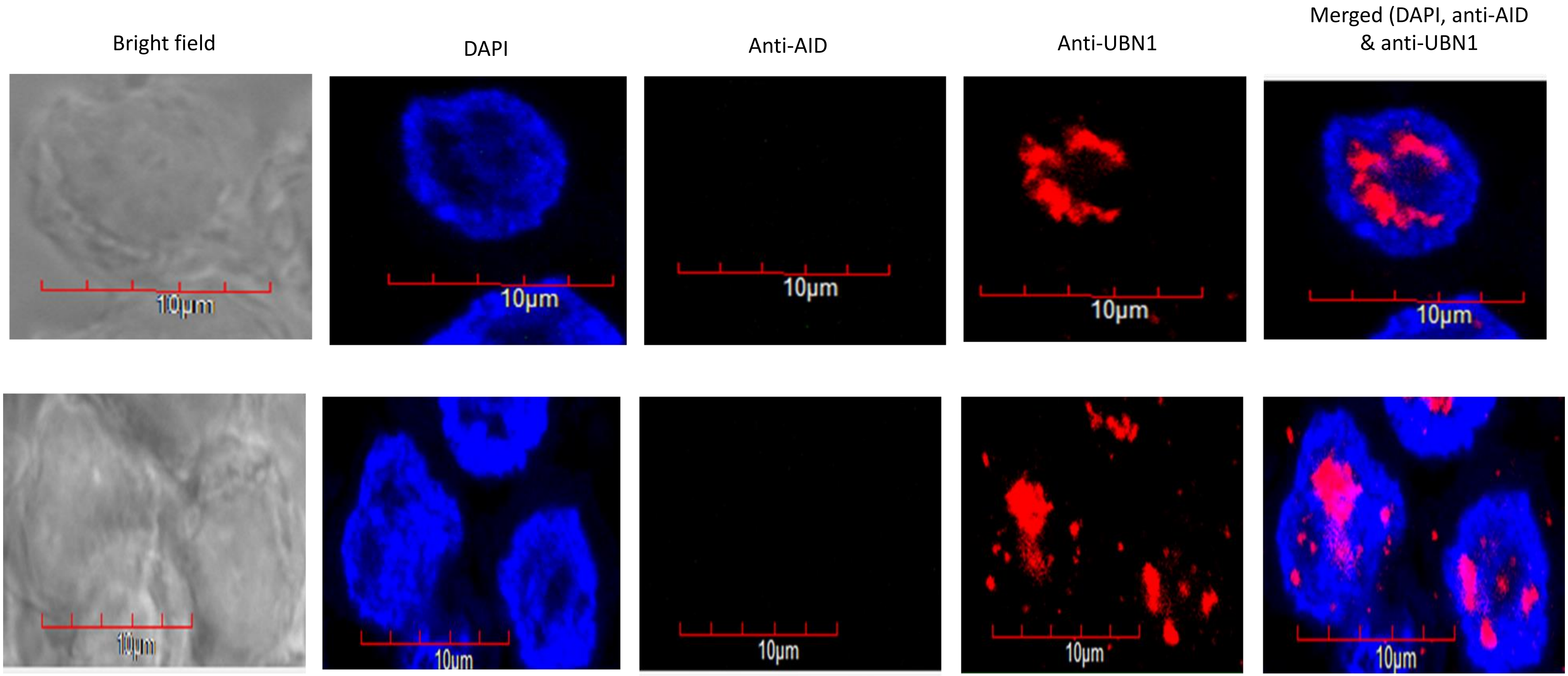

Fig. S13. Co-localization of AID with UBN1 in DT40 AID KO cells. Cells were fixed and double stained with monoclonal anti-AID (green) and polyclonal anti-UBN1 (red) antibodies. UBN1 is mostly inside the nucleus.

Fig. S14

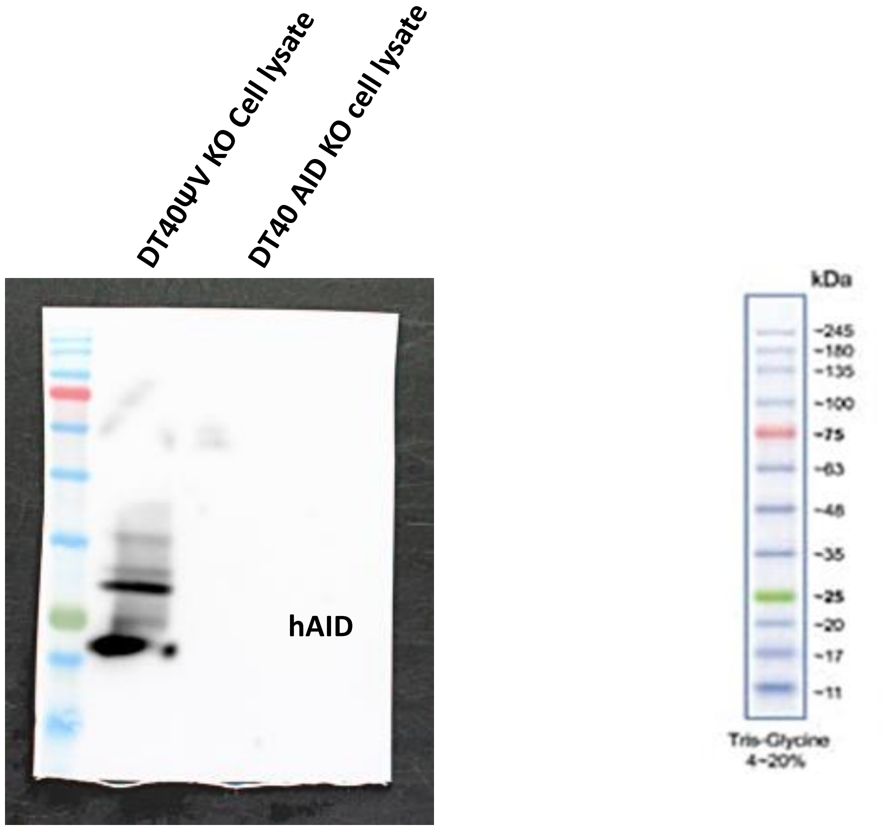

Fig. S14 Western blotting of DT40ΨV KO cell lysate as well as DT40 AID KO cells using anti-AID antibody.
